# Recyclable and Biodegradable Paper Coating with Functionalized PLA and PBAT

**DOI:** 10.1021/acsomega.4c11134

**Published:** 2025-03-14

**Authors:** Syeda Shamila Hamdani, Hazem M. Elkholy, Manal O. Alghaysh, Ian Wyman, Anibal Bher, Rafael Auras, Muhammad Rabnawaz

**Affiliations:** School of Packaging, Michigan State University, 448 Wilson Road, East Lansing, Michigan 48824-1223, United States

## Abstract

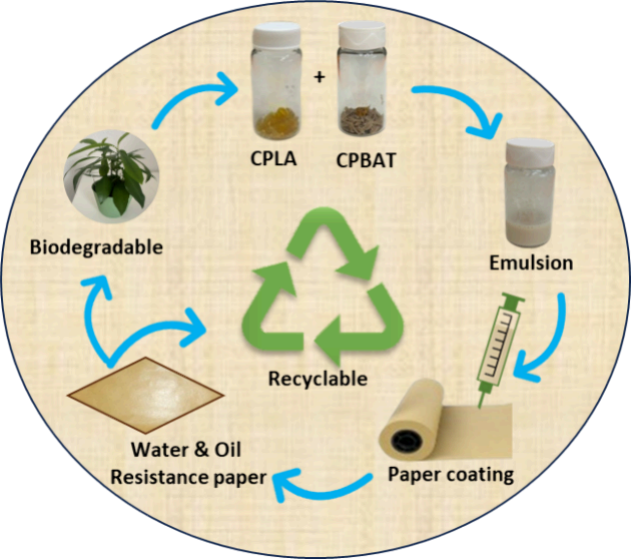

Conventional nonfunctional polyester-coated paper is suitable for packaging, but this coated paper is nonrecyclable. Herein, we report paper coating materials recyclable and industrially compostable using functionalized polyester blends. First, carboxylic acid-functionalized polylactic acid (CPLA) and carboxylic acid-functionalized poly(butylene adipate-*co*-terephthalate) (CPBAT) were synthesized. CPLA, either alone or as a blend of CPLA and CPBAT, was emulsified and then applied for paper coating. The coated paper was tested to evaluate its water and oil repellency, gas and moisture barrier, sealing, and mechanical properties index. The coated paper’s repulpability and recyclability were fully validated by certified methods. The compostability of the CPBAT- and CPLA-coated papers was also confirmed. This newly coated paper is PFAS- and persistent microplastics-free, and its recyclable nature fits into a circular economy approach.

## Introduction

Plastic is an economically promising material with excellent performance, making it suitable for single-use packaging.^1−3^ Plastic used in packaging corresponds to 40–46% of all plastic waste globally in 2006.^4,5^ However, the emergence of micro- and nanoplastics has puzzled scientists and the public about the future of plastic usage, particularly in single-use consumer packaging.^6−8^ Plastics used in packaging, either as solo materials or plastic-coated paper, degrade into tiny fragments, which are known as microplastics (i.e., <5 mm to <1 nm) when they leak into the environment.^9^ Microplastics can degrade into smaller nanoplastics (<1 μm). These microplastics can accumulate in microorganisms, animals, and humans. Microplastics have recently been found in human blood.^10,11^ In addition to microplastics, concerns are also related to per-and polyfluoroalkyl substances (PFAS), a commonly employed class of chemicals in paper packaging to impart water- and oil-repellent properties.^12^ On April 10, 2024, the US EPA issued, for the first time, national-level regulations for drinking water standards to safeguard communities from exposure to harmful PFAS, which are linked to cancers and can impact the kidneys, liver, and heart.^13^

When considering alternatives to plastic, paper is preferred for single-use consumer packaging due to its affordability, renewability, circularity, and biodegradability.^14^ However, transitioning to paper packaging has its challenges. Uncoated paper is hydrophilic, absorbs water, and is thus unfit to replace plastics.^15^ To overcome these challenges, plastics have traditionally been used to coat paper and enhance its water and oil repellency to address this issue. For instance, polyethylene (PE) has been widely used as a liner for paper substrates to improve water and oil repellency, thus enabling their use as paper coffee cups, disposable paper plates, and so forth.^16^ Wax has also been used, especially for low-temperature applications.^17,18^ However, both PE and waxes pose significant challenges to the recyclability and biodegradability of coated paper.

Our group has developed multiple strategies to improve the performance and environmental friendliness of paper coatings for packaging applications. These include a bilayer approach where the top layer is hydrophobic, and the bottom layer is hydrophilic,^19−21^ imparting water- and oil-repellent properties using polydimethylsiloxane (PDMS) as a nonfluorinated coating material,^22−24^ and a plant-based eco-friendly paper coating.^25^ These coatings are repulpable and, in many cases, biodegradable and recyclable.^26^ However, performance has yet to match that of commercial acrylic-laminated coated paper.

Using polyesters for paper coating to meet the performance and sustainability criteria is of practical importance. Polyhydroxyalkanoates (PHAs) offer good performance to coated paper and are biodegradable, but these polymers are relatively expensive, and PHA-coated paper is not recyclable.^27^ Polylactic acid (PLA) coatings can also provide paper with good performance,^28^ albeit with a brittle nature, and require thick coatings because of the melt-coating approach.^29^ As evident from recent literature, several waterborne coating formulations of PLA have been developed.^30−33^ Recent instances demonstrate an increasing interest in obtaining these formulations, employing PLA-coated paper is nonrecyclable because PLA cannot be separated from the paper under TAPPI recycling conditions.^34^

To address recyclability issues with polyester-coated paper, we reported recently a new system with a carboxyl-bearing poly(butylene adipate-*co*-terephthalate) (CPBAT) to enhance recyclability while offering remarkable performance that would be desirable for packaging.^35^ The method is cost-effective, as demonstrated by the TEA analysis of CPBAT-coated paper.^36^ However, PBAT is nonrenewable. We wanted to test the effect of biobased functionalized polyesters on the performance and recyclability of coated paper. We chose PLA as the biobased polymer for functionalization due to its biobased nature and its significantly lower carbon footprint compared to PBAT.^37^ In this study, carboxyl acid-bearing PLA (CPLA) polymers were synthesized and tested as paper coating material. CPLA was also blended with CPBAT and used as paper coatings. The coated papers were tested for water and oil resistance, thermal sealing capabilities, mechanical properties, biodegradation under composting, and recyclability.

## Experimental Section

### Materials

Poly(butylene adipate-*co*-terephthalate) (PBAT) was provided by Amcor Global (WI, USA). *Meso*-butane-1,2,3,4-tetracarboxylic dianhydride (MBTCA, purity >96%) was purchased from TCI (OR, USA). 1,4-Butanediol (purity 99%) and zinc acetate (purity 99.9%) were ordered from Sigma-Aldrich (MA, USA). l-lactide (purity 99%) was obtained from ASW MedChem, (NJ, USA). 1,10-Decanediol (purity 98%) and tin(II)-2-ethylhexanoate (purity 92.5–100%) were obtained from Sigma-Aldrich (MA, USA). Unbleached kraft paper was obtained from Uline (WI, USA). Corn starch and ammonium bicarbonate were purchased from Aldrich (MA, USA). All chemicals and materials were used as received without any further purification or modification.

### Methods

#### Synthesis of Carboxylic Acid-Functionalized Poly(lactic Acid) (CPLA)

CPLA was synthesized in two steps via a one-pot approach. In the first step, a 500 mL round-bottom flask was charged with 300 g of l-lactide (20 mol equiv), 18.14 g of 1,10-decanediol (1 mol equiv) and tin(II)-2-ethylhexanoate (0.5 wt % with reference to the weight of l-lactide). The obtained reaction mixture was stirred for 3 h under heating at 170 °C using a mechanical stirrer to produce PLA-diol with a yield of 99%.

During the synthesis of CPLA in the second step, the PLA- diol obtained in the first step was used without any further purification. Almost 318 g of PLA diol (1 mol equiv) was allowed to react with 20.60 g of MBTCA (1 mol equiv) at 170 °C under stirring using a mechanical stirrer, for 30 min to produce CPLA. Based on the actual recovery yields, the % yield of this step was 98%.

#### Synthesis of CPBAT

CPBAT was also synthesized in two steps using our reported procedure.^35^ In the first step, PBAT diol was synthesized via the reaction of commercial PBAT (200 g), 1,4-butanediol (8.80 g), and zinc acetate (1 wt %), in a 250 mL round-bottom flask, on heating at 200 °C. The mixture was continuously stirred using a mechanical stirrer for 6 h to produce PBAT-diol (98% yield).

In the second step, for the synthesis of CPBAT, the PBAT- diol obtained in first step was used without any further purification. For this step, almost 150 g of PBAT diol was placed in a 250 mL round-bottom flask before adding 16.50 g of MBTCA. The mixture was heated at 170 °C under stirring for 30 min (with a mechanical stirrer) to form CPBAT with a yield of 98%.

The % yield for both CPLA and CPBAT was assessed by weighing the final product, based on the percentage recovery of materials. ^1^H NMR was used to ensure the purity of CPBAT and CPLA and the lack of any residual reactants. Furthermore, no byproducts were generated as the reaction proceeded via chain growth polymerization during PLA-diol, PBAT-diol, and MBTCA.

#### Preparation of the Starch Solution

The base paper used in this study was prepared with the application of starch (5%) as the first layer. Before applying this starch layer, (5 wt %) starch stock solution was prepared by initially making a slurry of 5 g starch in 25 mL of deionized (DI) water under stirring to avoid any lumps. Once a homogeneous mixture was obtained, it was added to warm water (75 mL) in a 200 mL beaker and aluminum foil was used to cover the beaker. The mixture was continuously stirred and heated at 90 °C until a translucent solution was formed.

#### Preparation of CPLA and of the CPBAT Blend Emulsions

CPLA and CPBAT were cooled down to room temperature before being cut into smaller pieces, and then mixed in different weight ratios, as shown in Table 1. The obtained blend (total weight, 1.2 g) was emulsified with an aqueous solution of ammonium bicarbonate (0.4 g, 4 mL deionized water) at 85–90 °C for 10–15 min until a milky solution was obtained.

**Table 1:** Selected Formulations and Sample Codes Used in This Study^n^

**Abbreviated name**	**CPLA** (wt %)	**CPBAT** (wt %)
UKP^a^	-	-
SKP^b^	-	-
CPLA/CPBAT-100/0^c^	100	0
CPLA/CPBAT-90/10^d^	90	10
CPLA/CPBAT-80/20^e^	80	20
CPLA/CPBAT-70/30^f^	70	30
CPLA/CPBAT-60/40^g^	60	40
CPLA/CPBAT-50/50^h^	50	50
CPLA/CPBAT-40/60^i^	40	60
CPLA/CPBAT-30/70^j^	30	70
CPLA/CPBAT-20/80^k^	20	80
CPLA/CPBAT-10/90^l^	10	90
CPLA/CPBAT-0/100^m^	0	100

a Unmodified kraft paper.

b Kraft paper coated with 5% starch solution.

c Kraft paper coated with 100% CPLA emulsion.

d Kraft paper coated with an emulsion of a mixture having CPLA (90%) and CPBAT (10%).

e Kraft paper coated with an emulsion of a mixture having CPLA (80%) and CPBAT (20%).

f Kraft paper coated with an emulsion of a mixture having CPLA (70%) and CPBAT (30%).

g Kraft paper coated with an emulsion of a mixture having CPLA (60%) and CPBAT (40%).

h Kraft paper coated with an emulsion of a mixture having CPLA (50%) and CPBAT (50%).

i Kraft paper coated with an emulsion of a mixture having CPLA (40%) and CPBAT (60%).

j Kraft paper coated with an emulsion of a mixture having CPLA (30%) and CPBAT (70%).

k Kraft paper coated with an emulsion of a mixture having CPLA (20%) and CPBAT (80%).

l Kraft paper coated with an emulsion of a mixture having CPLA (10%) and CPBAT (90%).

m Kraft paper coated with an emulsion of 100% CPBAT.

n Note: All paper samples were initially precoated with a 5 wt % starch solution before applying the coating solution, except in the case of UKP, which was used as a control.

#### Paper Coating Procedure

Coated paper samples were developed with the application of 5 wt % starch as a base layer onto unmodified kraft paper using a coating machine (K303 Multi Coater) with rod number 8. The resultant 5% starch-coated papers were air-dried at an ambient temperature for 24 h before applying a waterborne coating as a top layer. The waterborne emulsion solutions were applied onto starch-coated kraft paper with a silicon spatula followed by initially drying in an oven at 100 °C for 10 min before it was subjected to quick drying at 160 °C until a shiny surface appeared. Oven-dried coated paper samples were further air-dried at ambient temperature for 24 h before other analyses. Table 1 provides information about the final formulations and abbreviated names of samples used in this study.

### Characterization

#### Attenuated Total Reflectance Fourier-Transform Infrared (ATR-FTIR) Analysis

FTIR analysis of the material used in this study and paper samples both before and after coating was carried out using a Jasco FTIR-6600 spectrometer (Maryland, USA). The samples were analyzed with a total number of 16 scans over a 4000–500 cm^–1^ range.

#### ^1^H NMR Analysis

Proton nuclear magnetic resonance (^1^H NMR) spectroscopy (500 MHz, Varian 7600-AS, USA) was used to analyze the synthesis of the CPLA and CPBAT samples. Each polymer sample (5 mg) was dissolved in 0.8 mL of deuterated chloroform (CDCl_3_) to prepare the sample for ^1^H NMR characterization.

#### Basis Weight and Thickness

The thicknesses of the unmodified and coated paper samples were measured using a digital micrometer (Testing Machine Inc., DE, USA). Ten random places on the paper samples were used to measure the thickness of each sample, which were described as an average value in μm. The basis weight was measured using samples with 12 × 2.5 cm^2^ dimensions and reported as the mass per square meter per the standard ASTM D646 protocol. Each sample was weighed before coating and after coating. The difference between the weight of each sample before and after coating was recorded, and divided with the sample area in square meters. The difference in basis weight before and after coating the paper samples was recorded as coating load expressed in grams per square meter.

#### Scanning Electron Microscopy (SEM)

Paper samples were coated with a thin layer of platinum (4 nm) before SEM analysis using a Q150T ES turbo-pumped sputter coater (Quorum Technologies, England) under argon purging. The SEM analysis of the platinum-coated samples was then carried out with a JEOL 6610 SEM system (JEOL Ltd., Japan).

#### Brightfield Microscopy

Brightfield-transmitted light images were used to characterize emulsions. All images were recorded using a Nikon Eclipse Ni upright microscope, configured with a10x Plan Apo objective (NA 0.3) and a Nikon DS-Fi2 color camera.aThe particle size was measured using Nikon NIS-Elements AR Imaging Software (version 5.42.03).

#### Water Resistance

A Cobb sizing tester (Büchel BV Inc. Utrecht, Netherlands) was used to record the Cobb600 values following the TAPPI protocol (T441 om-09). A 100 cm^2^ paper sample was brought in contact with 100 mL of DI water for 600 s (10 min). The sample was weighed before and after water was poured onto it. The difference in their weight (g) was divided by the area in square meters, and results were reported as Cobb600 values, which were expressed in units of grams per square meter (g/m^2^).

Droplet tests were performed to observe the water penetration into each paper sample, which indicates water resistance against liquid water (samples having higher water resistance showed greater resistance against water penetration). A droplet of 0.1 mL DI water was applied to the paper sample during each of these tests. Pictures were recorded at various intervals to observe the droplet penetration into the paper, including before the application of the droplet, 5 min after the application of the water droplet, and after the removal of the droplet.

#### Water Vapor Transmission Rate (WVTR) Measurements

A Permatran-W system (Model 3/34, Mocon Inc., MN, USA) was used to record the WVTR at 23 °C and 50% relative humidity (RH) to record water resistance against liquid vapors. Paper samples measuring 2 × 2 cm^2^ were fixed in an aluminum mask sheet to prepare the samples for testing. A 0.5 cm^2^ open hole in the aluminum mask sample was left to expose the paper surface to incoming water vapor. Before WVTR analysis, all samples were preconditioned for 1 h to achieve the targeted conditions set conditions (23 °C and 50% RH) inside the machine.

#### Oil Resistance

The oil resistance of paper samples was analyzed via kit test following a standard TAPPI (T559 pm-96) method. The kit test was conducted using a series of kit test solutions (numbered 1 through 12) with varying proportions of castor oil, *n*-heptane, and toluene. The test liquids have varying surface tensions and viscosities. Those with a higher kit rating are more “aggressive” or likely to become absorbed by the paper sample than those with lower kit numbers. The kit test results were expressed as kit ratings, which were in the range of 0–12. A kit rating of 0 corresponds to a sample having the lowest oil resistance, while a kit rating of 12 corresponds to the maximum oil resistance. During the test, a 0.05 mL liquid droplet from various kit solutions was applied onto coated paper for 15 s, which was removed with chem wipes. The appearance of a dark spot after applying the liquid with a certain kit number onto the tested paper surface indicated that the paper sample had failed the test with that particular liquid. A specific kit number is assigned when its corresponding kit solution does not produce any dark spot on the tested paper surface after residing on it for 15 s.

In addition, oil droplet tests were performed. The methodology for the oil droplet behavior study was the same as that employed for the water droplet test, except that pure castor oil was used in place of water droplets.

#### Thermogravimetric Analysis (TGA)

TGA analysis was conducted using a Q-50 thermogravimetric analyzer (TA Instruments, DE, USA), and measurements were made on a temperature range of 10–600 °C. A sample weighing 8 mg in a standard pan was heated at a 10 °C/min ramping rate. At a flow rate of 40 mL/min, the test was conducted in a nitrogen atmosphere. The first derivative of the TGA curves was examined to record the derivative thermogravimetric (DTG) curves.

#### Molecular Weight Determination

The average molecular weights were determined using size exclusion chromatography (SEC) was along with dispersity values of PLA-diol, CPLA, neat PBAT, PBAT-diol, and CPBAT. A Waters Gel Permeation Chromatograph (Waters Associates Inc. MA, USA) was used coupled with an autosampler (Waters 717plus) and an isocratic pump (Waters 1515). The samples were prepared by dissolving 10 mg of solid material in 5 mL of HPLC-grade tetrahydrofuran (THF; Sigma-Aldrich, USA) and transferred to a vial using a PTFE-GF syringe filter (pore size = 0.45 μm and diameter = 13 mm). THF was used as the mobile phase solvent with a flow rate of 1 mL min^–1^. A series of Styragel columns including HR-4, HR-3, and HR-2 columns (300 mm × 7.8 mm I.D.) were connected to the SEC system along with a refractive index detector (Waters 2414) kept at a constant temperature of 35 °C. The calibration standard was a polystyrene standard (Shodex STANDARD SM-105, Tokyo, Japan) and the *M*_w_ (weight-average molecular weight), dispersity values along with *M*_n_ (number-average molecular weight), were calculated using Waters Breeze 2 software. Values are reported relatively to a polystyrene calibration curve.

#### Water Contact Angle (WCA) Measurements

An AST VCA 2500XE Video Contact Surface Inspection Goniometer Fuji 611847 (AST Products, Inc. MA, USA) was used to analyze the WCAs. During each of these tests a 5 μL DI droplet was placed on a paper sample with the help of a motorized syringe. Images were recorded at 30 s and 5 min intervals following the droplet application to calculate the WCAs with the help of a VCA Optima system. The value of the WCAs was reported as the mean value of data recorded in triplicates.

#### Mechanical Properties

A 5565 Universal Instron Testing Machine (Instron, MA, USA) was employed in exploring the tensile strength, Young’s modulus, and elongation at the break of each paper sample following a TAPPI (T 494) standard procedure with the help of the Bluehill universal software version 4.25 (Instron, MA, USA). Paper samples with precise dimensions of 1 × 4 in.^2^ were prepared with a JDC precision sample cutter. Every sample was tested while being stretched at a steady rate of 12.5 mm/min and with a grip separation of 25 mm.

The Ring Crush Test (RCT) analysis was conducted using the TAPPI (T882) standard protocol. Samples with specific dimensions of 0.5 × 6 in.^2^ were prepared using a TMI precision sample cutter F215/225 (Testing Machine Inc., OH, USA). Tests were conducted in both the machine direction (MD) and the cross directions (CD) utilizing TMI, Emerson’s Model 1210 Crush Tester (MA, USA).

#### Thermal Sealing Properties

Before the thermal seal strength measurements, samples with specific dimensions of 1 × 4 in.^2^ were prepared and sealed at 400 °F with a 4 s sealing time. A bar sealer (SENCORP, MA, USA) with a seal width of 0.4 in. was used for the sealing process All the sealed samples were preconditioned at 25 °C and 50% RH for 24 h before testing of their seal strength. After this seal strength was recorded with the help of a 5565 Universal Instron Testing Machine (Instron, MA, USA) at a rate of 12 in./min and a grip separation of 1 in, following the standard ASTM F88/F88M-2 protocol. Bluehill universal software version 4.25 was also used for these tests. The seal strength was recorded as the force (*N)* required to break seal, at maximum load and breakpoint.

#### Repulpability

The first step in evaluating the recyclability of paper-based samples is to explore their repulpability. The repulpability was evaluated via a certified protocol “Tappi Voluntary Standards Part-I Repulping” and samples of the CPLA/CPBAT-100/0 and CPLA/CPBAT-0/100 systems were selected for this test. Twenty-five g of coated paper was trimmed down to small strips measuring 1.1/4 (width)x 4 (length) in.^2^ and was soaked in 1500 mL of warm water for 4 h at a temperature of 125 ± 10 °F. Following the soaking process, the paper was repulped using a Waring blender with nonsharp blades operated at 15,000 rpm. The repulping in the blender was performed for 4 min. After repulping, the fibers were deflaked for up to 5 min with a British Disintegrator. The total volume in the British Disintegrator was maintained up to 2000 mL with a pH of 7 (±0.5 pH), operating at 3000 rpm, and the temperature was maintained at 125 ± 10 °F. Subsequently, the pulp solution was poured onto a flat screen with 0.010-in. holes and attached to running water. The fibers were separated based on their size from impurities and large fibers. Net accepts, and net rejects were collected and dried in a laboratory oven at 105 °C for the time period of 12 h. Once dried, both the net rejects and net accepts were weighed, and the % yield of fiber recovery was calculated using eq 1:
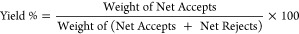
1

To pass the repulping test, the threshold limit is 85% yield.

#### Recyclability

The recyclability test for samples of CPLA/CPBAT-100/0 and CPLA/CPBAT-0/100 was conducted following a standard procedure known as the “Tappi Voluntary Standards Part-II Repulping”. A coated paper sample was mixed with UKP in 80% and 20% ratios during the procedure, respectively. The mixed paper was repulped using repulping test mentioned above. The obtained pulp (net accepts) hand sheets were prepared, pressed, and subsequently, air-dried for 24 h. The air-dried handsets were tested for various properties to determine whether or not the tested samples were recyclable. All the properties were tested following their respective TAPPI protocols. In detail, a water drop penetration test was performed following the TAPPI (T831) protocol and other properties included the coefficient of friction by TAPPI (T815), stickies count by TAPPI (T277), burst strength by TAPPI (T403), and short span compression strength (STFI) by the TAPPI (T831) standard protocol. The properties of coated paper samples were compared to those of base paper to evaluate the recyclability of each tested sample. As benchmarks, we tested commercial PLA- and commercial PBAT-coated kraft paper which each had a base layer that was prepared with 5 wt % starch as a base layer. These control samples were prepared by taking commercial PLA and PBAT, dissolving them in chloroform and then coating them onto paper.

#### Compostability

The compostability of samples was evaluated under standardized conditions by performing a thermophilic test in simulated composting conditions at 58 ± 2 °C and 50 ± 5% RH in a direct measurement respirometer (DMR). CO_2_ evolution was measured during the 120 days of the test, and its value was used to calculate the % biodegradation of each sample based on the theoretical carbon content of the material evaluated (Table S1, Supporting Information). Samples were tested as 1 by 1 cm for paper and coated paper, while the material used for coating was tested as small irregular pieces. Mature compost was obtained from the MSU Composting Facility and sent to an external lab to analyze physicochemical parameters (Table S2, Supporting Information). Prepared samples were mixed with conditioned mature compost in a relation 1:50 by wt. Samples were run in triplicate, and results are reported as average values plus the calculated standard error. Compost, samples, and their preparation in the bioreactors, as well as required periodic tasks when the test ran, are reported elsewhere.^38^

## Results and Discussion

**Scheme 1:**
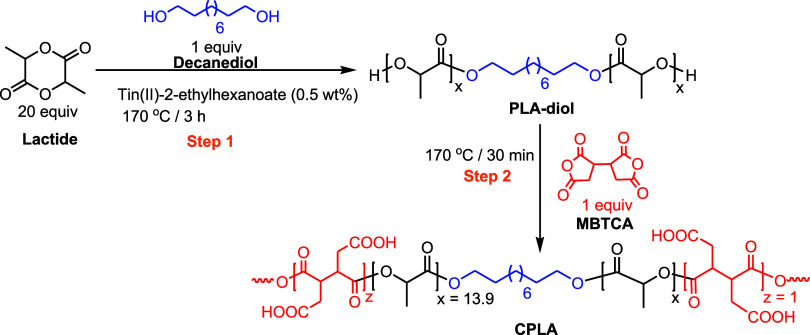
Synthetic Pathway Leading to the Formation of CPLA The first step is the formation of PLA-diol, and the second step involves the synthesis of CPLA.

Scheme 1 depicts the synthesis of CPLA, which was synthesized in two steps. First, PLA was synthesized via the ring-opening polymerization of l-lactide. Here, 1,10-decanediol was used as an initiator that yielded PLA-diol. The OH groups of PLA-diol were subsequently reacted with the dianhydride groups of MBTCA to afford CPLA. In the first step, we used twenty-mole equivalents of l-lactide and one-mole equivalent of 1,10-decane diol to produce PLA-diol with 20 lactide units (40 lactic acid units). Subsequently, an equivalent mole ratio of OH moieties of PLA-diol and anhydride moieties of MBTCA was reacted to create CPLA.

**Figure 1:**
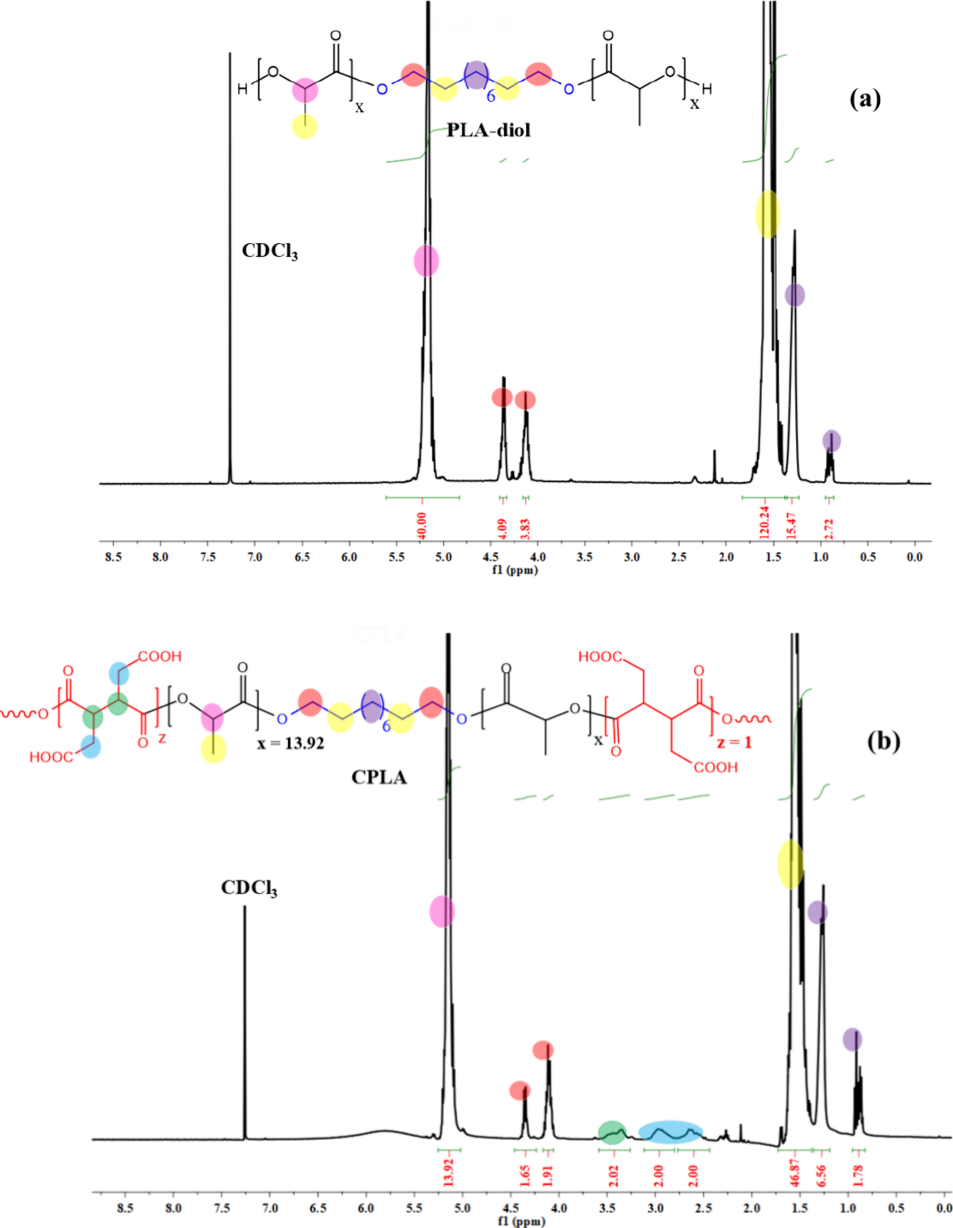
^1^H NMR spectra of PLA-diol (a) and CPLA (b).

Both PLA-diol and CPLA were characterized by ^1^H NMR spectroscopy, as shown in Figure 1. The spectra showed multiplets corresponding to the methylene groups at 0.85–1.27 ppm, and multiplets corresponding to the methyl and methylene protons of the lactide and decane moieties at 1.46–1.57 ppm. Additionally, the spectrum of CPLA (Figure 1b) revealed multiplet corresponding to the methylene protons of MBTCA adjacent to carboxylic groups in the range 2.50–3.08 ppm and the methine protons of MBTCA adjacent to ester groups at 3.25–4.63 ppm. Moreover, the methylene protons of the decane in the vicinity of oxygen atoms and the methine protons of lactide appeared at 4.06–4.35 and 5.15 ppm, respectively. Additionally, the CPLA copolymer composition was calculated based on the ^1^H NMR spectrum by comparing the integration of two methine protons of lactide (−CH−) and methylene protons of the opened structure of MBTCA (−CH_2_−) which are 13.9:1.0. Based on this ratio, the respective mole percentages are 93.3% (x) for PLA and 6.7% (z) for COOH, reflecting the proportions of each component relative to the entire copolymer mixture. Similarly, CPBAT was obtained by first creating low molecular weight PBAT-diol after the degradation of high molecular weight PBAT with 1,4-butanediol, and then the resulting PBAT-diol was chain extended with MBTCA using our recently reported method^35^ presented in Scheme S1 and characterized by ^1^H NMR spectroscopy, Figure S1, Supporting Information. The *M*_n_ of PLA-diol was calculated from the ^1^H NMR spectrum (Figure 1a) and found to have a value of ∼3000 amu using a reported method.^39^

The average molecular weights of PLA-diol, C-PLA, PBAT-diol, and CPBAT were determined via SEC and are shown in Table 2. PLA diol had *M*_n_, *M*_w_, and Dispersity as 2.3 kDa, 3.3 kDa, and 1.45, respectively. After reaction with dianhydride, the produced CPLA had *M*_n_, *M*_w_, and Dispersity as 11.42 kDa, 19.60 kDa, and 1.72, respectively. PBAT-diol had *M*_n_, *M*_w_, and dispersity values of 6.1 kDa, 7.3 kDa, and 1.18, respectively. After the reaction with dianhydride, the produced CPBAT had 8.5 kDa, 13.9 kDa, and 1.64 as its *M*_n_, *M*_w_, and dispersity values, respectively. SEC data suggests the successful formation of CPLA and CPBAT.

**Table 2:** Average Molecular Weights and PDI Values of PLA-diol, CPLA, Neat PBAT, PBAT-diol, and CPBAT^a^

**Polymer**	*M*_*n*_**(kDa)**	*M*_*w*_**(kDa)**	**Dispersity**
PLA-diol	2.3	3.3	1.45
CPLA	11.42	19.60	1.72
PBAT-diol	6.1	7.3	1.18
CPBAT	8.5	13.9	1.64

a Note: PBAT-diol and CPBAT analysis are based on reference.^35^

**Figure 2:**
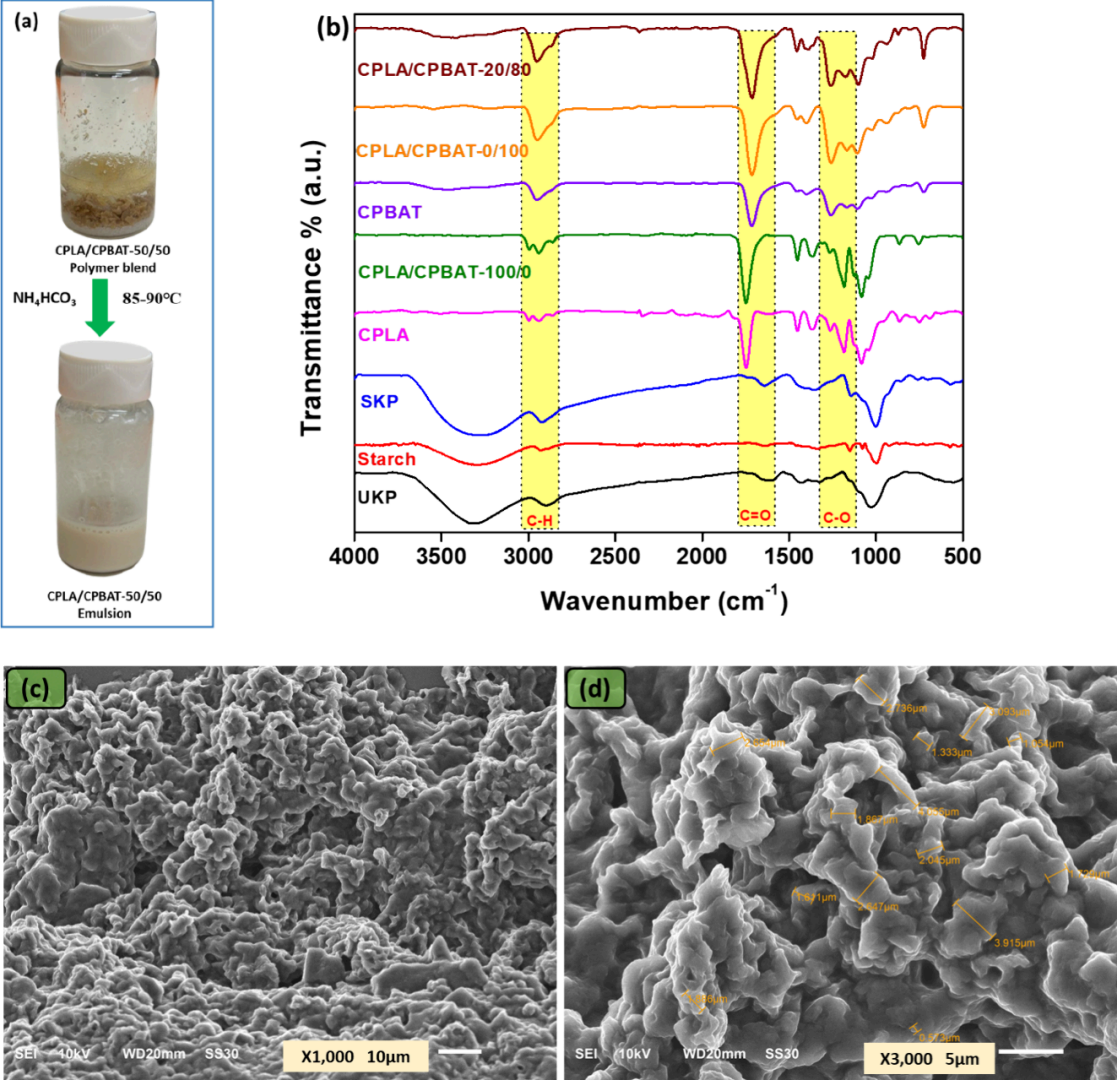
(a) Ionization of a CPLA and CPBAT blend, yielding a waterborne emulsion that was subsequently employed as a coating material and (b) ATR-FTIR spectra of unmodified kraft paper (UKP) and solid materials employed for the preparation of coating solutions. These spectra include those of neat starch (starch), starch-coated kraft paper (SKP), the solid polymer CPLA, paper coated with an emulsion of 100% CPLA material (CPLA/CPBAT-100/0), the solid polymer CPBAT, paper coated with an emulsion of 100% CPBAT material (CPLA/CPBAT-0/100), and coated paper prepared with a blend of CPLA and CPBAT having 20% CPLA and 80% CPBAT in a dry weight ratio (CPLA/CPBAT-20/80). SEM images of CPLA/CPBAT-50/50) emulsion (c) at 10 μm scale and (d) at 5 μm scale showing various sizes of dissolved polymer particles.

These polymers were neutralized with ammonium bicarbonate to prepare emulsions from CPLA and CPLA/CPBAT blends. The emulsions were formed at 90 °C in 10–15 min. The formation of a milky solution was an indication of successful emulsion formation. A summary of the emulsification process (Scheme S2, Supporting Information) and photographs of the polymer blend and the obtained emulsion are shown in Figure 2. The obtained emulsions were applied onto starch coated kraft paper via the rod coating method. The paper was quickly dried at 160 °C after initially drying at 100°C for 10 min. After heat treatment, the paper was conditioned and stored at room temperature for 24 h prior to characterization and properties investigation.

The CPLA/CPBAT-50/50 was characterized by SEM, as shown in Figure 2c,d. The results indicate that particle sizes vary between 0.57 to 4.06 μm and are uniformly distributed. The particles are nonspherical, a typical pattern one would expect from the dispersion of a premade polymer, in this case, CPLA/CPBAT-50/50, when dispersed in water. Optical microscopy analysis has also been used to characterize emulsions, and particle size has been measured, as shown in (Figure S2a–c, Supporting Information).

### ATR-FTIR Analysis

The synthesized CPLA and CPBAT were characterized via ATR-FTIR spectroscopy along with neat starch, coated paper, and unmodified paper samples, as shown in Figure 2b. In the spectrum of UKP, a broad band around 3300–3400 cm^–1^ indicated the presence of cellulose-free hydroxyl groups. A similar band was visible in the spectrum of SKP, the starch-coated paper sample, due to the presence of hydroxyl groups of the starch (and cellulose of paper). In the spectrum of the polymer CPLA, aliphatic C–H stretching peaks appeared around 2931 and 2990 cm^–1^.^40^ In addition, a carbonyl stretching vibration band appeared at 1746 cm^–1^, C–H bending bands at 1449 and 1363 cm^-1^, and a C–O stretching band appeared at 1180 cm^–1^. Similar peaks were found on the coated paper CPLA/CPBAT-100/0, indicating the successful application of coating material containing CPLA. The neat polymer CPBAT was also characterized via ATR-FTIR spectroscopy and exhibited a C–H stretching vibration band at 2934 cm^–1^, the carbonyl stretching (C = O) band was observed at 1710 cm^–1^ as a prominent peak, and a C–O–C stretching band appeared at 1240 cm^–1^.^41^ The coated paper sample CPLA/CPBAT-0/100 coated with an emulsion comprised solely of CPBAT was also characterized and showed similar peaks to those exhibited by the CPBAT polymer, indicating the application of CPBAT onto its surface. The coated paper prepared with the blend of CPLA and CPBAT having 20% CPLA and 80% CPBAT in a dry weight ratio (CPLA/CPBAT-20/80) exhibited prominent peaks corresponding to CPBAT on its surface due to the presence of CPBAT as the major component of the coating material. In general, the ATR-FTIR data confirmed the successful synthesis of CPLA and CPBAT as well as the successful application of these polymer-based coatings onto the surfaces of paper substrates.

### Basis Weight and Thickness

The thickness (μm), basis weight (g/m^2^), and coating load (g/m^2^) of the coated and unmodified kraft paper samples were investigated, and the obtained data is shown in Table 3. The thickness of unmodified kraft paper (UKP) was increased from 180.2 ± 3.7 to 204.0 ± 6.1 μm after applying a starch layer (SKP). There were further increases in thickness for the samples coated with an emulsion comprised solely of CPLA (i.e., CPLA/CPBAT-100/0) up to 228.1 ± 9.1 μm and the thickness increased to 234.5 ± 12.7 μm for the sample that was coated with an emulsion comprised solely of CPBAT (i.e., CPLA/CPBAT-0/100). Paper coated with a blend of CPLA and CPBAT also showed an increase in thickness to 232.8 ± 12.4 μm for the sample CPLA/CPBAT-20/80. The basis weight was also increased for the blend sample CPLA/CPBAT-20/80 (to 176.5 ± 1.0 g/m^2^), which was higher than the basis weight of the unmodified kraft paper (127.6 ± 0.6 g/m^2^). Similarly, the value for coating load for the blend sample CPLA/CPBAT-20/80 was found to be 46.5 ± 1.0 g/m^2^ concerning the reference UKP (0 g/m^2^) and SKP (9.4 ± 1.1 g/m^2^) samples. These findings indicate that an increase in coating load was noticed by applying a starch layer onto the unmodified kraft paper, with a further increase being achieved with the subsequent application of an emulsion layer. It is noteworthy that the CPLA/CPBAT-100/0 coating solution was less viscous, resulting in an overall lower coating load compared to the CPLA/CPBAT-20/80 and CPLA/CPBAT-0/100 solutions. In the future, more detailed studies on the relationship between coating load and performance will be required.

**Table 3:** Thickness, Basis Weight, and Coating Load of Coated Paper Samples in Comparison to the Unmodified Paper Sample

**Sample no.**	**Material thickness (μm)**	**Basis weight** (g/m^2^)	**Coating loading** (g/m^2^)
UKP	180.2 ± 3.7	127.6 ± 0.6	0
SKP	204.0 ± 6.1	137.1 ± 1.1	9.4 ± 1.1
CPLA/CPBAT-100/0	228.1 ± 9.1	165.4 ± 0.8	37.3 ± 0.8
CPLA/CPBAT-20/80	232.8 ± 12.4	174.1 ± 2.1	46.5 ± 1.0
CPLA/CPBAT-0/100	234.5 ± 12.7	176.5 ± 1.0	48.9 ± 2.1

### Scanning Electron Microscopy Analysis (SEM)

**Figure 3:**
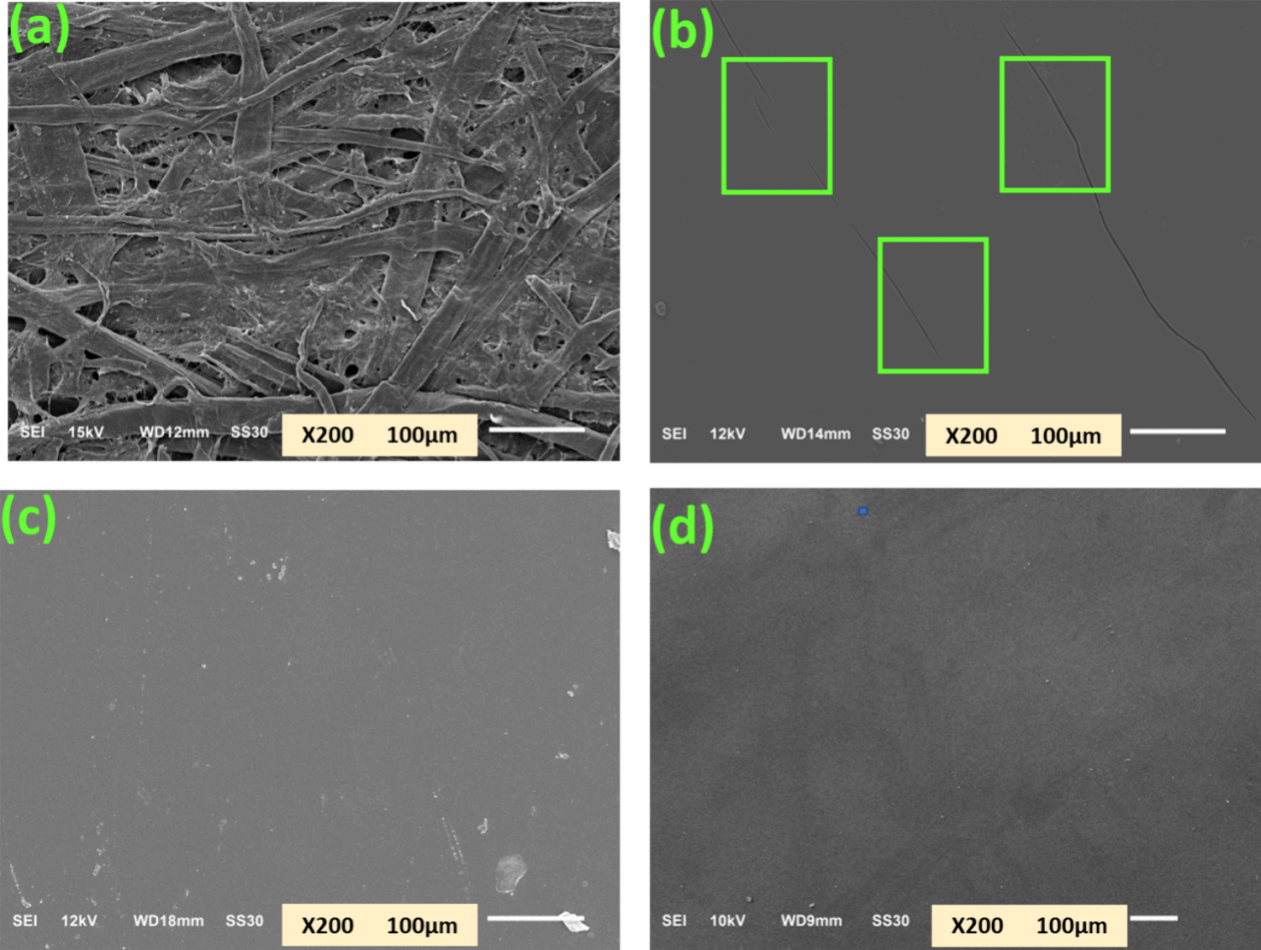
SEM images (200×) of: (a) unmodified kraft paper (UKP), (b) paper coated with an emulsion comprised solely of CPLA material (CPLA/CPBAT-100/0), (c) paper coated with the blend of CPLA and CPBAT having 20% CPLA and 80% CPBAT in a dry weight ratio (CPLA/CPBAT-20/80), and (d) paper coated with the emulsion of 100% CPBAT material (CPLA/CPBAT-100/0). Note: The green rectangular boxes in panel (b) denote the cracks that appeared on the surface of CPLA/CPBAT-100/0 coated paper.

The increased barrier properties of the coated paper samples can be well explained by the SEM images shown in Figure 3, which display the surface morphologies of the paper samples before and after coating. It can be seen (Figure 3a) that UKP has many holes on its surface, which account for its poor barrier properties (later discussed). These holes are nicely covered by applying the CPLA layer using its emulsion, and the resultant coated paper CPLA/CPBAT-100/0 (Figure 3b) shows a very smooth surface. However, due to brittleness associated with CPLA, cracks are formed on CPLA-coated paper surfaces, such as those shown in the green boxes in Figure 3b. These cracks may lead to water, oil, and water vapor absorption. In contrast, the sample prepared by applying an emulsion-based coating comprised of a blend of CPLA and CPBAT (i.e., CPLA/CPBAT-20/80, Figure 3c) possesses a smooth surface without any cracks due to the flexible nature of CPBAT. A similar smooth surface without any cracks can also be seen in the SEM image of the paper sample coated by the emulsion comprised solely of CPBAT (i.e., CPLA/CPBAT-100/0, Figure 3d). These images show that the brittleness of CPLA-coated paper can be reduced by blending CPLA with CPBAT to obtain high barrier-coated paper samples.

### Water Resistance

**Figure 4:**
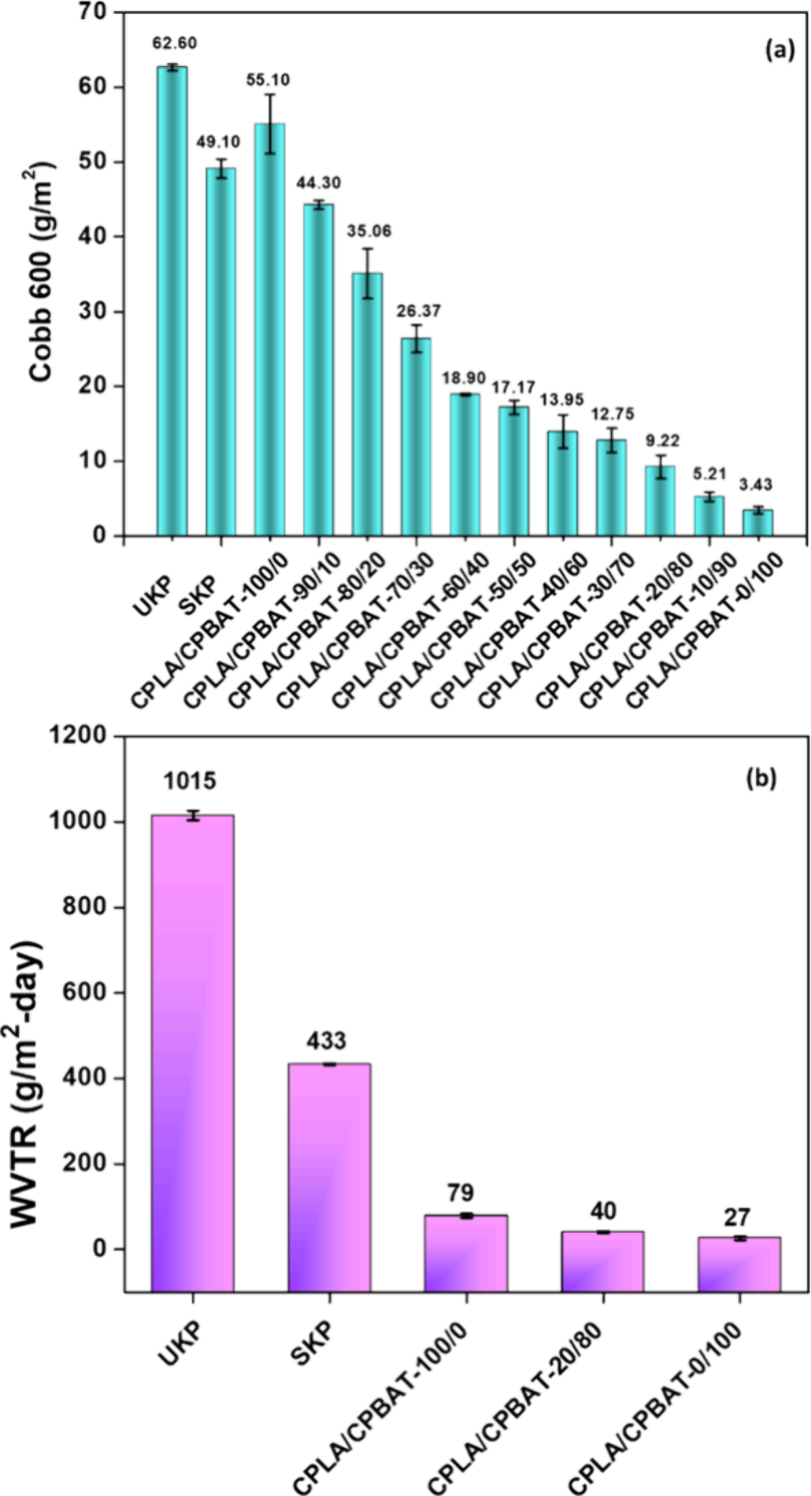
(a) Cobb600 values (g/m^2^) of UKP, SKP, and emulsion-coated paper samples that were prepared using various weight ratios of CPLA and CPBAT. (b) WVTR (g/m^2^-day) values of coated paper samples compared to unmodified kraft paper tested at 23 °C and 50% relative humidity.

Applying hydrophobic polymers onto the surfaces of paper substrates can help improve the water-resistance of the resultant-coated paper samples so that they can have applications in the packaging industry. In this study, the water resistance was increased with the application of various blends of CPLA/CPBAT using various weight ratios of CPLA and CPBAT. The water resistance was measured against liquid water and water vapor. In particular, the water resistance against liquid water was explored by measuring the Cobb600 values of coated paper samples compared to those of UKP, and the results are shown in Figure 4a.

The result showed that unmodified kraft paper showed a very high Cobb600 value of 62.60 ± 0.43 g/m^2^ due to the hydrophilic nature of cellulose fibers. Applying a starch layer onto kraft paper led to a slight decrease in the Cobb600 value to 49.10 ± 1.27 g/m^2^ due to its good masking performance. Still, the water resistance was only modestly improved due to the hydrophilic nature of starch. The SEM data (Figure 3) discussed earlier revealed that the surface was nicely covered after applying the CPLA emulsion. Still, cracks had formed on the surface of the coated paper, and consequently, the Cobb600 value of CPLA/CPBAT-100/0 remained the same. In contrast, the introduction of CPBAT along with CPLA increased the water resistance of the resultant coated paper, as was demonstrated by a decreasing Cobb600 value. The sample CPLA/CPBAT-20/80 (with 80% CPBAT and 20% CPLA in the emulsion) showed a low Cobb600 value of 9.22 ± 1.58 g/m^2^, which is ideal as we are looking for an economic approach to achieve the minimum Cobb-600 value while still having practical applicability and utilizing a minimal amount of CPBAT. Meanwhile, the sample CPLA/CPBAT-0/100 that was coated with the emulsion comprised solely of CPBAT showed a Cobb600 value of 3.43 ± 0.46 g/m^2^, which shows that CPBAT has a much higher tendency to impart water repellency than CPLA. Overall, there was an 85% reduction in the Cobb600 value for the sample CPLA/CPBAT-20/80, which had a low Cobb600 value compared to UKP while incorporating CPBAT.

The water resistance against liquid water was also studied by recording the water contact angles (WCAs) on various samples (Figure S2d, Supporting Information). Selected samples were analyzed by applying a water droplet onto each sample’s surface, and the WCA was recorded 30 s after the application of the droplet. The water droplet remained in place for up to 5 min, and the WCA was rerecorded to explore the droplet’s behavior on the surface of the paper sample. The result showed that the WCA of UKP was 36.05 ± 1.20° at 30 s, which dropped to zero after 5 min and was accompanied by the appearance of a dark spot, indicating that the water had been completely absorbed by UKP, thus demonstrating that the paper had very poor water resistance. The WCA of the sample CPLA/CPBAT-100/0 was 59.30 ± 0.70° at 30 s, which dropped to 47.45 ± 0.91° after a 5 min interval and was accompanied by the appearance of a light stain on the paper. This decline in the WCA and the formation of a light stain can be attributed to the cracks in this coating, which thus also resulted in poor water resistance. In contrast, the WCA of sample CPLA/CPBAT-0/100 was higher than those of all tested samples (i.e., 76.89 ± 0.01°), demonstrating that this sample had high water resistance. The incorporation of CPBAT was found to enhance the water resistance of the CPLA-containing coatings upon blending, as shown by the paper sample CPLA/CPBAT-20/80, which had a WCA value of 69.45 ± 1.90° at 30 s that declined modestly to 61.20 ± 1.69° at 5 min. The WCA value observed on the coated paper samples after 5 min was still higher than that observed on unmodified paper at 30 s. This finding was consistent with the Cobb600 data and demonstrated that the water resistance of the coated paper samples had been enhanced significantly.

To explore the visual effect of water droplets on coated paper surfaces in comparison to UKP, a water droplet was placed on the surfaces of various paper samples, and images were recorded after the passage of 5 min, followed by removal of the water droplet using Kim Wipe (Figure S3, Supporting Information). The stains appeared due to water absorption. The results showed a dark spot appeared on the UKP surface, and a light spot was visible on the sample SKP and CPLA/CPBAT-100/0 surface, indicating that these three samples had poor water resistance. In contrast, the surfaces of the samples CPLA/CPBAT-0/100 and CPLA/CPBAT-20/80 remained intact after the water droplets had been wiped away, suggesting that these two samples possessed excellent water resistance.

To assess the water resistance against water vapors, the WVTR values for various paper samples were measured, as shown in Figure 4b. The testing conditions were 50% RH and 23 °C. The WVTR data closely paralleled the Cobb600 values. The sample that was prepared by blending CPLA and CPBAT (CPLA/CPBAT-20/80) exhibited a WVTR of 40.50 ± 2.44 g/(m^2^-day), which was far less than that of UKP, which had a WVTR value of 1014.92 ± 11.40 g/(m^2^-day). Overall, there was a drastic decrease in the WVTR values of coated paper compared to that of UKP up to 95%, indicating that the coated paper samples possessed high barrier properties against water vapor.

### Oil Resistance

**Figure 5:**
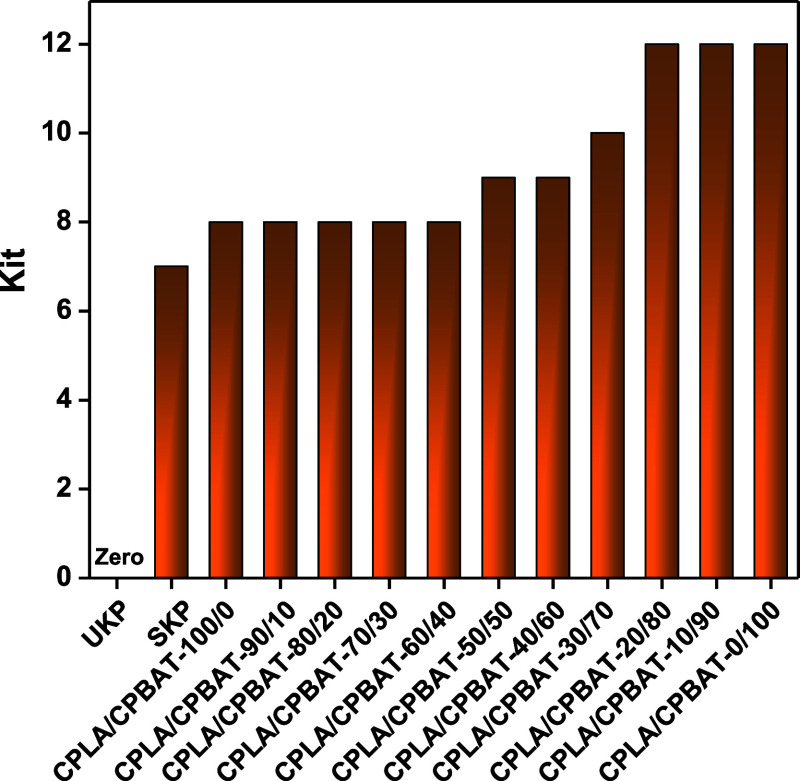
Kit ratings of unmodified kraft paper and all coated paper samples using various CPLA and CPBAT weight ratios.

The oil resistance of coated paper samples was measured by recording their kit numbers. The kit rating corresponds to the highest number of kit solutions that remain on the tested paper for 15 s after it has been applied without leaving any dark trace. Before the oil resistance tests, kit solutions were prepared with kit numbers ranging from 1 to 12. A higher kit rating indicates that a tested sample has higher oil resistance and vice versa. For example, a kit rating of 0 is assigned to a surface when it fails a test performed with a test liquid with a kit number of 1, thus indicating that the surface has very poor oil resistance. Figure 5 shows that sample UKP possesses a kit rating of 0, indicating that it has very poor oil resistance due to its porous nature so that oil can be quickly taken up by the UKP sample. The paper sample SKP showed better oil resistance due to the oleophobic nature of starch, with a kit rating of 7. The blend samples incorporating CPBAT in CPLA showed a slight increase in their kit ratings up to 8, while the CPLA/CPBAT-50/50 showed a slight further increase in their kit ratings up to 9. There was a further increase in the kit rating for the sample CPLA/CPBAT-30/70 up to 10, while the kit rating was increased to highest kit value 12 (on the kit rating scale) for the sample CPLA/CPBAT-20/80. This data demonstrates that the incorporation of CPBAT in CPLA during blending increases the oil resistance of the resultant samples as the cracks that appear on coated papers due to inherent brittleness of CPLA has been masked by CPBAT increasing content in coating solution validated by SEM data (Figure 3).

The visual effect of castor oil droplets placed on the surfaces of paper samples was also explored similarly to the corresponding tests with water droplets described earlier. The results (Figure S4, Supporting Information) revealed that castor oil droplets were quickly taken up by UKP, leaving dark brown spots. This behavior indicated that the UKP had poor oil resistance. In contrast, none of the coated paper samples exhibited dark spots on their surfaces after 5 min had elapsed following the application of the droplets, thus demonstrating that the coated paper samples have improved oil resistance compared to UKP’s.

### TGA Analysis

The thermal stabilities of the coated paper samples were investigated via TGA analysis, as shown in Figure S5, Supporting Information. To explore the effect of the coating materials on the thermal stabilities of the paper samples, TGA analysis of neat coating materials was also carried out. The TGA plots obtained for UKP, and various coated paper samples are shown in Figure S5a, Supporting Information, and those obtained for the solid coating materials are shown in Figure S5b, Supporting Information. Similarly, DTG curves for both paper samples (uncoated and coated) are presented in Figure S5c, Supporting Information, while DTG curves for the coating materials are shown in Figure S5d, Supporting Information. The results indicate that major degradation of UKP occurred ∼380 °C, while all the coated paper samples showed high thermal stability in the range of 355 to 385 °C which shows the maintenance of thermal stability of substrate even after the application of coating material on paper. Figure S5b, Supporting Information, shows that neat starch material decomposed at ∼324 °C, CPLA decomposed at ∼284 °C and CPBAT decomposed at ∼396 °C. Figure S5b,d, Supporting Information, shows that CPLA had lower thermal stability than other solid materials. Still, the thermal stability of the resultant coated paper increased when it was coated onto paper after it had been blended with CPBAT. For example, the CPLA/CPBAT-20/80 paper sample underwent major degradation at ∼357 °C. These findings indicate that the coated papers developed may be suitable for contact with hot food items in the food and beverage packaging industry

### Thermal Sealing Properties

Selected coated paper samples were tested to explore their thermal seal strength using our previously reported method.^42,38^ In this scenario, the force required to break each seal at maximum load and breakpoints were recorded, as shown in Figure S6, Supporting Information. The results revealed that all the tested samples exhibited high seal strength comparable to a commercial control (Eco-Shield paper). Among the tested samples, CPLA/CPBAT-0/100 showed the highest seal strength (i.e., 10.10 ± 1.23 N) at maximum load compared to Eco-Shield paper (10.20 ± 0.66 N). The seal strength values for other samples, such as CPLA/CPBAT-100/0 and CPLA/CPBAT-20/80 at maximum load, were also very close (i.e., 9.09 ± 0.95 and 9.53 ± 1.24 N, respectively). A similar trend was found for the seal strength at the break point, except for sample CPLA/CPBAT-0/100, which showed a higher seal strength at its breakpoint (i.e., 3.80 ± 0.16 N) compared to that of Eco-Shield paper (1.77 ± 0.30 N). These thermal sealing results suggest that the coated paper samples have significant potential for application in packaging such as manufacturing paper bags, fast food boxes, etc., without requiring any additional adhesives.

### Mechanical Properties

**Figure 6:**
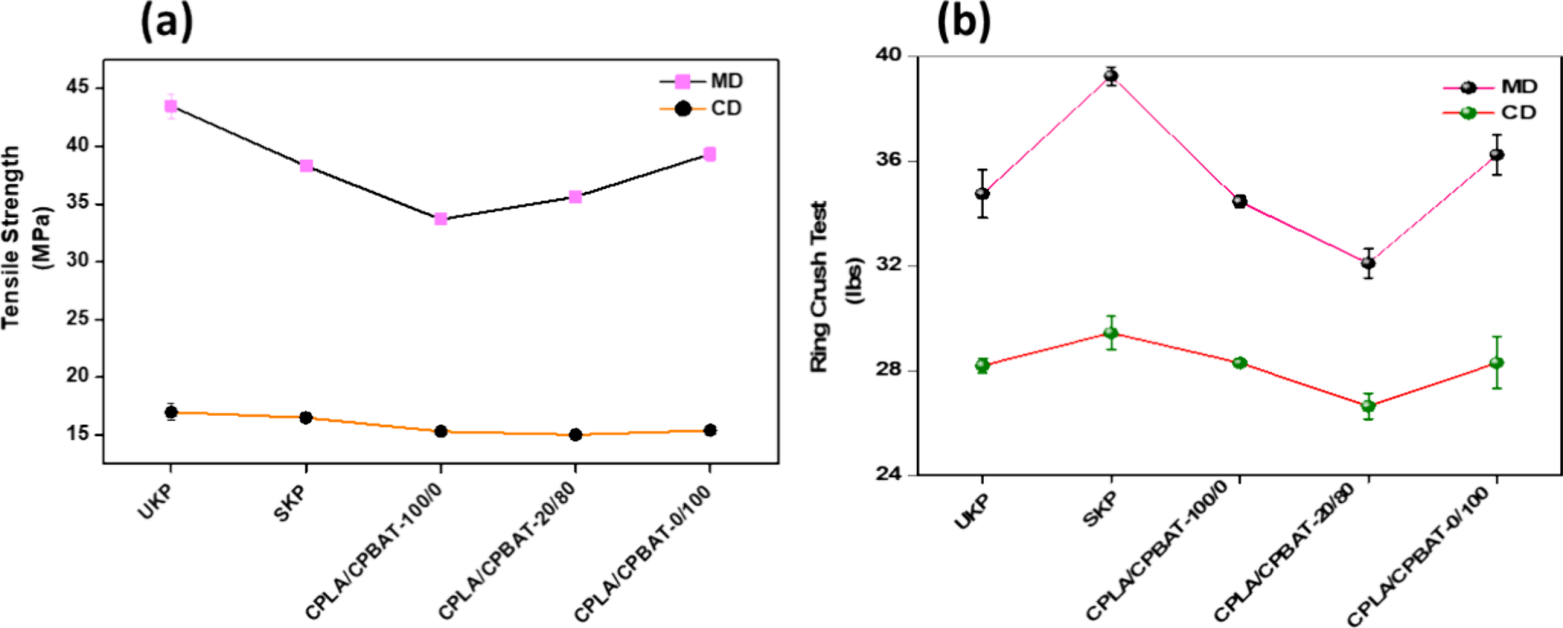
Tensile strength (a), and ring crush test performances (b) of unmodified paper kraft paper in comparison to various coated paper samples.

The durability of a package refers to its resilience to maintain strength and function over a period of time when it is subjected to different environmental conditions, which mainly depends upon its mechanical properties. The mechanical properties of a package have a direct impact on its ability to shield its contents from outside impacts from packing through the distribution chain. Figure 6 shows the data obtained from different mechanical tests that were performed to analyze the strength of designed coated paper samples, including tensile strength, Young’s modulus, elongation at break and ring crush test. Figure 6a shows that in the MD, a 10–22% decrease in the tensile strengths was observed following the application of coating material. For example, SKP showed a tensile strength of 38.30 ± 0.46 MPa, which was lower than that of UKP, which had a higher tensile strength of 43.50 ± 1.05 MPa. There were further decreases in the tensile strengths of other coated papers, to 33.66 ± 0.49, 35.63 ± 0.49, and 33 ± 0.58 MPa for CPLA/CPBAT-100/0, CPLA/CPBAT-20/80, and CPLA/CPBAT-0/100, respectively. These results show that the blending of CPBAT with CPLA shows some decrease but does not significantly change the tensile strengths of the resultant coated papers. A similar trend has been found for Young’s modulus (Figure S7a, Supporting Information) values. In MD, the sample CPLA/CPBAT-100/0 showed the maximum decrease of 36% and sample CPLA/CPBAT-0/100 showed decrease in young’s modulus value up to 15% only compared to UKP. The blend sample CPLA/CPBAT-20/80 showed value of 2043 ± 40.41 MPa with maximum deviation from UKP up to 25% (2770 ± 17.32 MPa). In the CD, all the samples showed a decrease in Young’s modulus from 18 to 29% of original UKP value. % Elongation at break values were increased for all the coated samples up to 51% in both MD and CD in contrast to UKP (Figure S7b, Supporting Information).

The RCT values show the ability of a package to withstand a compressive force before it collapses under the applied pressure, with a higher RCT value corresponding to a stronger package (Figure 6b). The coated paper sample CPLA/CPBAT-20/80 showed RCT values of 32.10 ± 0.56 lbs in the MD and 26.65 ± 0.49 lbs in the CD, while the corresponding values for UKP were 34.75 ± 0.91 lbs in the MD and 28.20 ± 0.28 lbs in the CD. These results revealed that coated paper prepared by blending CPBAT and CPLA had retained its RCT values by up to 95% both in the MD and the CD. In general, it is observed that the coated paper samples maintain 80–95% of the most of original mechanical properties exhibited by the UKP.

### Recyclability

**Figure 7:**
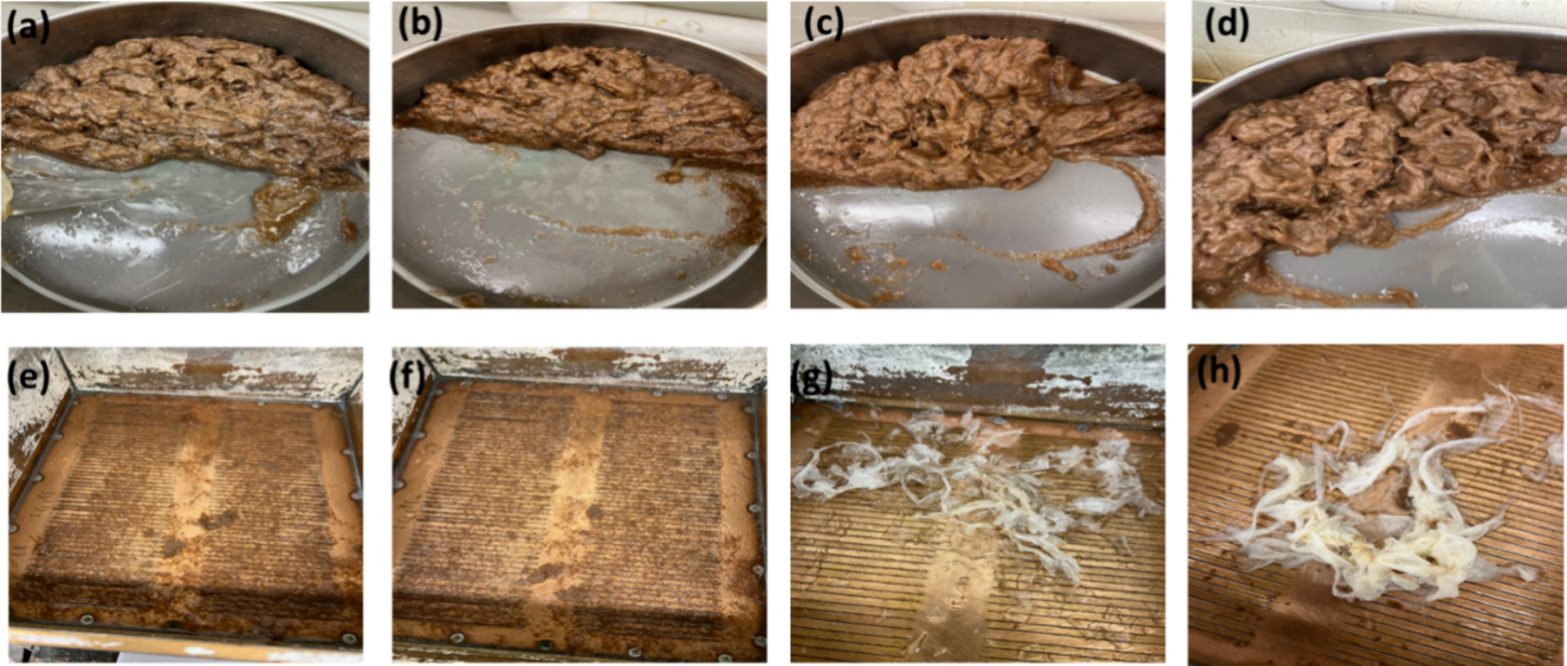
Screen accepts obtained for samples of CPLA/CPBAT-100/0 (a), CPLA/CPBAT-0/100 (b), commercial PLA-coated paper (c), and commercial PBAT-coated paper (d). Screen rejects obtained for samples of CPLA/CPBAT-100/0 (e), CPLA/CPBAT-0/100 (f), commercial PLA-coated paper (g), and commercial PBAT-coated paper (h).

The recyclability of paper products is important for stimulating sustainability, lowering environmental impact, and preserving economic resources by offering a closed-loop system, which reduces the demand of virgin wood pulp for paper manufacturing. To meet this growing need for a closed-loop approach, we tested the repulpability and recyclability of our coated paper samples CPLA/CPBAT-0/100 and CPLA/CPBAT-100/0 to determine whether our blends of CPLA and CPBAT could yield recyclable systems. Initially, the repulpability was tested and the recyclability of samples that had met the repulpability criteria was subsequently investigated. Papers coated with commercial PLA and commercial PBAT were used as controls. The complete repulping data is shown in Table 4 and images of screen accepts and screen rejects of pulp are shown in Figure 7. The results revealed that paper samples coated with commercial PLA and commercial PBAT failed the lab-scale repulpability tests. Also, for PLA and PBAT coated paper, the coating film became wrapped around the blender, so that it was necessary to disassemble the blender and remove the coating films (Figure S8, Supporting Information). In contrast, the samples coated with CPLA and CPBAT blends met the criteria of 85% fiber recovery, thus passing the certified repulpability test. In comparison to CPLA/CPBAT-100/0, which had an 86.46% fiber recovery, the paper sample CPLA/CPBAT-0/100 shown a higher fiber recovery of up to 94.44%. These findings have demonstrated that, as the paper coated with each of these polymers separately is repulpable, the coated paper produced by blending the CPLA with CPBAT to obtain a coating material would also be repulpable.

**Table 4:** Percentage Yields/Fiber Recovery Rates Obtained for Samples CPLA/CPBAT-100/0, CPLA/CPBAT-0/100, and Commercial Controls During the Repulping Procedure^a^

		**Sample Code**			
**Content**	**Unit**	**CPLA/CPBAT-**100/0	**CPLA/CPBAT-**0/100	**Commercial PLA-coated paper**	**Commercial PBAT-coated paper**
Sample charged	g	23.10	23.30	24.89	25.06
Screen reject	g	1.13	2.80	3.39	3.64
Screen accepts	g	19.22	17.88	15.64	19.19
Yield of sample	wt %	94.44	86.46	82.19	81.03
Pass/Fail		Pass	Pass	Fail	Fail

a Note: Repulping data for sample CPLA/CPBAT-0/100 are already reported in reference.^35^

Both CPLA/CPBAT-100/0 and CPLA/CPBAT-0/100 were subjected to recyclability testing following a standard protocol. The pulp was converted to hand sheets which were screened for basis weight, coefficient of friction, water drop penetration, stickies count and burst strength analysis. Results revealed that both the samples also passed the laboratory-scale recycling, as no significant deviation in the tested properties (less than 15%) was found between base paper used as a control and tested coated papers, except for the stickies count which was less than 30%. An overall summary of the recycling data is shown in Table 5.

**Table 5:** General Properties Studied to Evaluate the Recyclability of Paper Samples CPLA/CPBAT-100/0 and CPLA/CPBAT-0/100^a^

**Performance**	**Unit**	**Base paper (UKP)**	**CPLA/CPBAT-**100/0	**CPLA/CPBAT-**0/100
Basis weight	g/m^2^	100	103	101
Coefficient of Friction	Degrees	32.4	34.0	30.5
Water drop penetration	Seconds	2.0	2.3	2.3
Stickies	Counts	35	45	31
Burst strength	lb/inch^2^	19.8	20.9	23.8
	Index value	0.198	0.203	0.235
Short Span Compression Strength (STFI)	lb/inch	4.51	4.89	4.61
	Index value	0.045	0.047	0.046

a Note: Recycling data for sample CPLA/CPBAT-0/100 are already reported in reference.^35^

### Biodegradation in Simulated Composting Conditions

**Figure 8:**
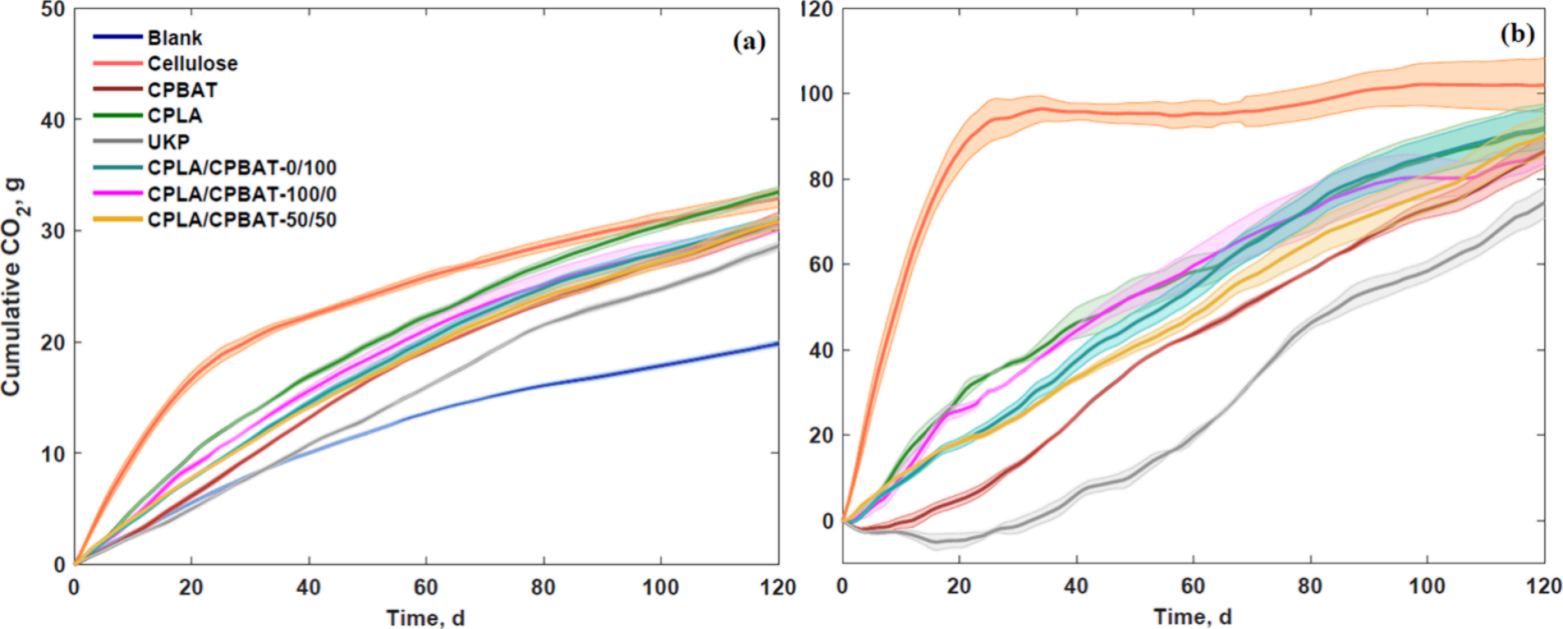
(a) Cumulative CO_2_ (g), and (b) biodegradation (%) for blank (compost), cellulose (positive control), CPBAT, CPLA, UKP, CPLA/CPBAT-0/100, CPLA/CPBAT-100/0, CPLA/CPBAT-50/50 samples.

Figure 8a shows the CO_2_ evolution and biodegradation for the evaluated samples. The positive control cellulose showed a fast initial biodegradation reaching a plateau phase at around 30 days of testing (Figure 8b). Reference material (UKP) for modified samples shows the lowest biodegradation after 120 days with around 80%. Unmodified kraft paper has been evaluated previously for biodegradation showing a higher value (over 100%) after 120 days underlying the challenge of a wood fiber made material evaluation under composting conditions due to different lignin composition.^38^ Furthermore, it was previously shown the role of cellulosic made material and its contribution to developing priming effect in compost media.^44^ Coated paper samples and material used for coating in general showed biodegradation with values around 90% after 120 days meeting the requirements of evolving 90% by 180 days of biodegradation since there was a characteristic trend of the samples of still going up in terms of their biotic phase without the appearance of a visible plateau phase at 120 days of testing. However, in the case of the coated paper samples, a remaining fraction was observed after 120 days indicating that likely a less industrially compostable fraction associated with the lignin fraction of the paper is remaining (reported as around 20–25% for commercial kraft paper).^45,46^ This is not detrimental from a biological point of view since lignin that is not converted to CO_2_ can be degraded to a stable humic substance that is sometimes desirable as an end product for soil restoration.^47^ Thickness of samples (around 180 μm for UKP and over 200 μm for coated paper) could potentially play a differential role when evaluating biodegradation.

## Conclusions

CPLA and its blends with CPBAT were successfully synthesized and converted into waterborne emulsions. Blends in which CPLA was 20 wt % or less offered good water resistance. For example, Cobb600 values reached as low as 9.22 ± 1.58 g/m^2^ for sample CPLA/CPBAT-20/80. The coated paper also exhibited excellent oil resistance, reaching 12/12 on the kit rating scale. SEM analysis confirmed that CPLA-coated paper bore cracks due to the brittle nature of CPLA, and thus, CPLA alone had low water resistance. The addition of CPBAT into CPLA reduced the brittleness, and cracks disappeared. Both CPLA and CPLA/CPBAST blend-coated paper gave good thermal sealing properties, as well as retained 80–95% of the original mechanical properties.

Overall, coated paper, as well as materials used for coating, show consistent biodegradation under simulated composting conditions until 120 days of testing and with an active biotic phase afterward, potentially meeting the requirements for biodegradation in thermophilic industrial composting conditions. The remaining fraction of coated paper samples at the end of the test indicates a likely high fraction of lignin content for unbleached kraft paper. On the other hand, the material used for coating showed an acceptable biodegradation performance under composting, highlighting its potential as an environmentally friendly alternative to commercial nonbiodegradable coating papers. Our CPLA- and CPBAT-coated paper also passed certified recycling tests, while the commercial PLA- and PBAT-coated papers are nonrecyclable and failed recycling test. Thus, this study offers a significant advancement toward a renewable, circular, and economical approach to paper coating.

## Supporting Information Available

Additional details of the experiments including: synthetic pathway leading to CPBAT (Scheme S1); ^1^H NMR spectra of CPBAT (Figure S1); synthetic pathway leading to ionic CPLA and ionic CPBAT using ammonium bicarbonate (Scheme S2); brightfield-transmitted light, magnified images analysis of emulsions and water contact angle analysis (Figure S2); water droplet test (Figure S3); oil droplet analysis (Figure S4); TGA and DTG analysis (Figure S5); thermal sealing analysis (Figure S6); Young’s modulus and elongation at break (Figure S7); photograph showing failure of the repulping test with commercial PBAT-coated paper (Figure S8); carbon content of samples evaluated during biodegradation (Table S1); physicochemical parameters of compost used for biodegradation test (Table S2); stress–strain curves for uncoated and coated paper samples (Figure S9); DSC analysis (Figure S10); and stability of emulsions visual analysis (Figure S11) (PDF)

## Notes

H.M.E. is on leave from the Department of Chemistry, Faculty of Science, Tanta University, Tanta, 31527, Egypt.
