# Supplementary material for: Recyclable and Biodegradable Paper Coating with Functionalized PLA and PBAT
Source: ACS Omega. 2025 Mar 14;10(11):11483–97. doi: 10.1021/acsomega.4c11134 (PMC11948148; doi:10.1021/acsomega.4c11134)
Supplement: Supplementary file 1 — ao4c11134_si_001.pdf [file ao4c11134_si_001.pdf]

Supporting Information

For

Recyclable and Biodegradable Paper Coating with Functionalized PLA and PBAT

Syeda Shamila Hamdani[#a], Hazem M. Elkholy[#a], Manal O. Alghaysh[a], Ian Wyman[a], Anibal Bher[a], Rafael Auras[a] and Muhammad Rabnawaz[a]*

[a]School of Packaging, Michigan State University, 448 Wilson Road, East Lansing, Michigan, 48824-1223, United States

*Corresponding author: rabnawaz@msu.edu

[#]Authors with equal contribution

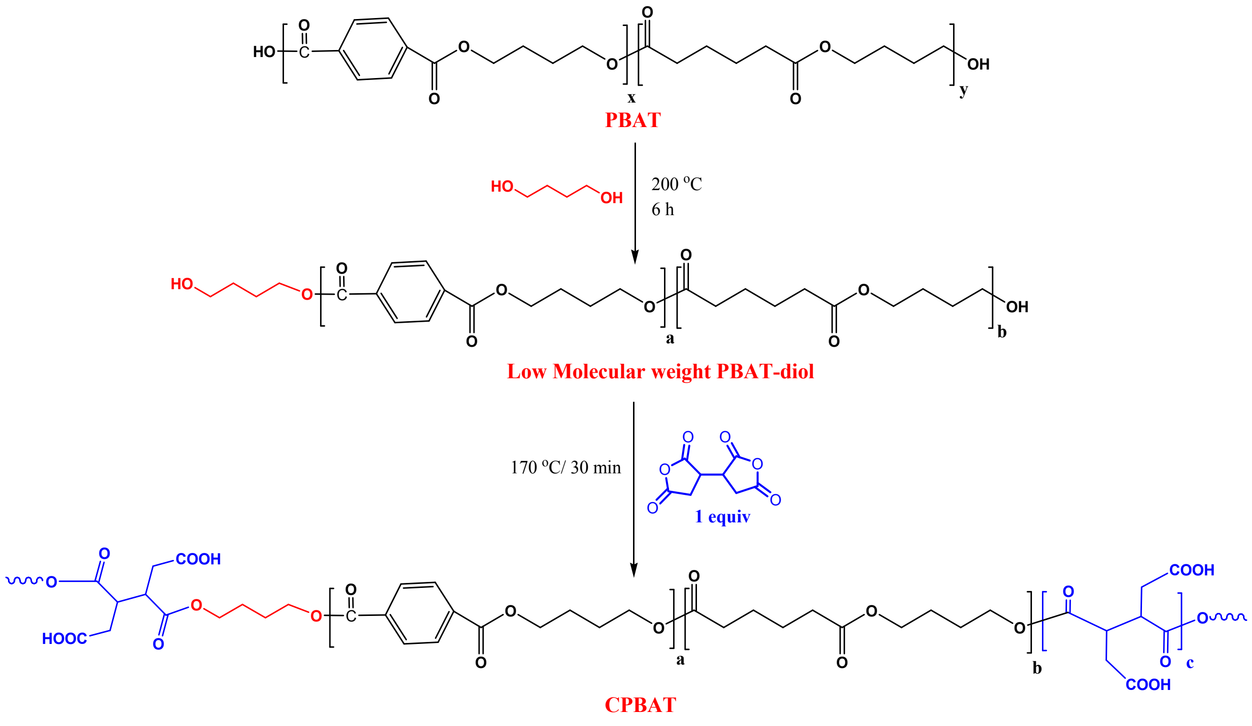

Scheme S1. Synthetic pathway leading to CPBAT. The wavy lines denote polymer chains

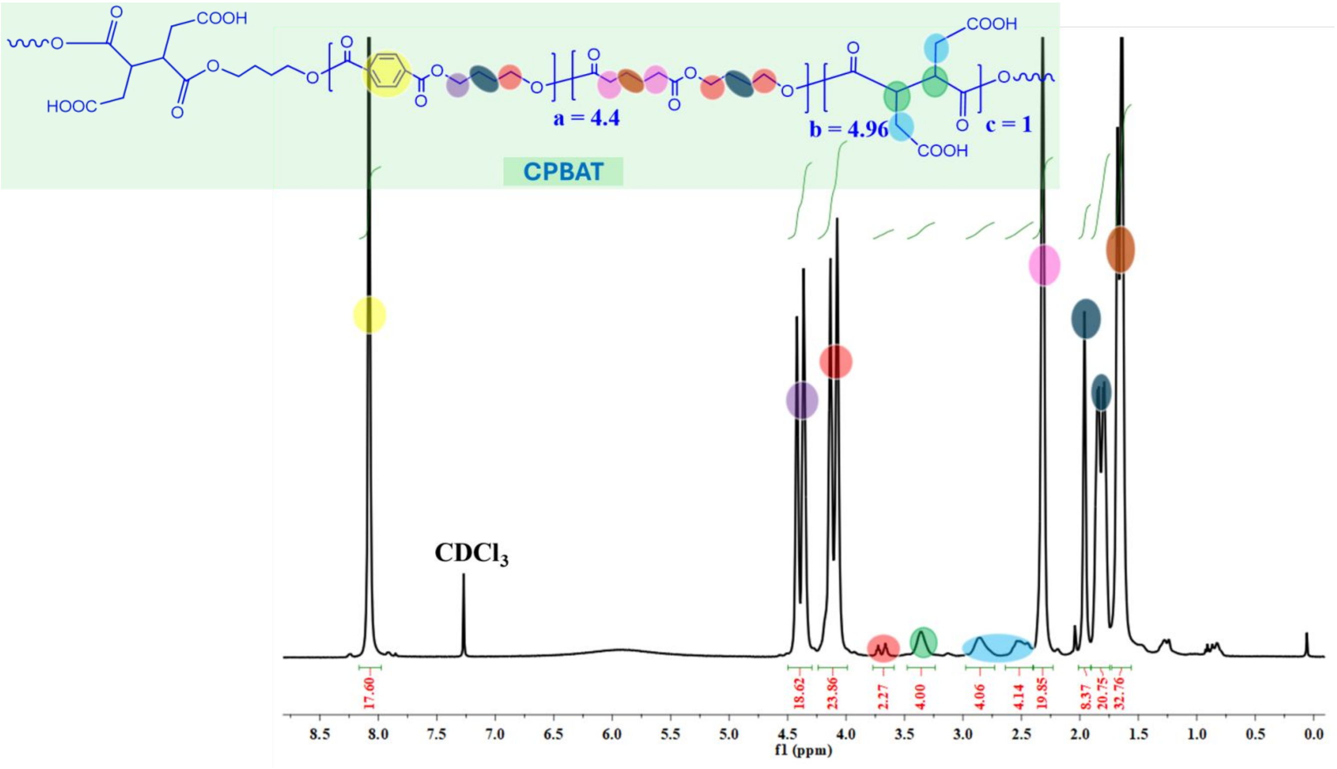

Figure S1. ^{1}H-NMR spectra of CPBAT

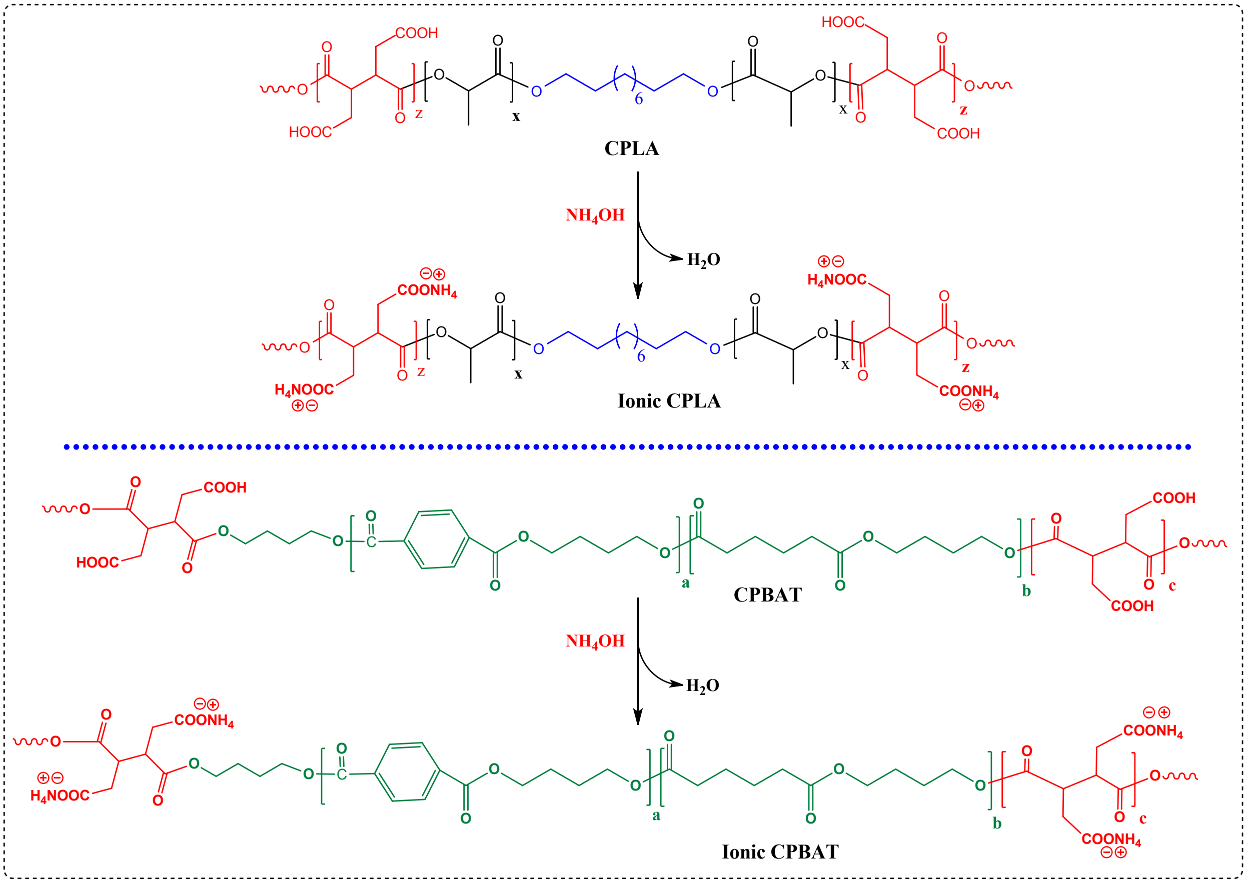

Scheme S2. Synthetic pathway leading to ionic CPLA and ionic CPBAT using ammonium bicarbonate.

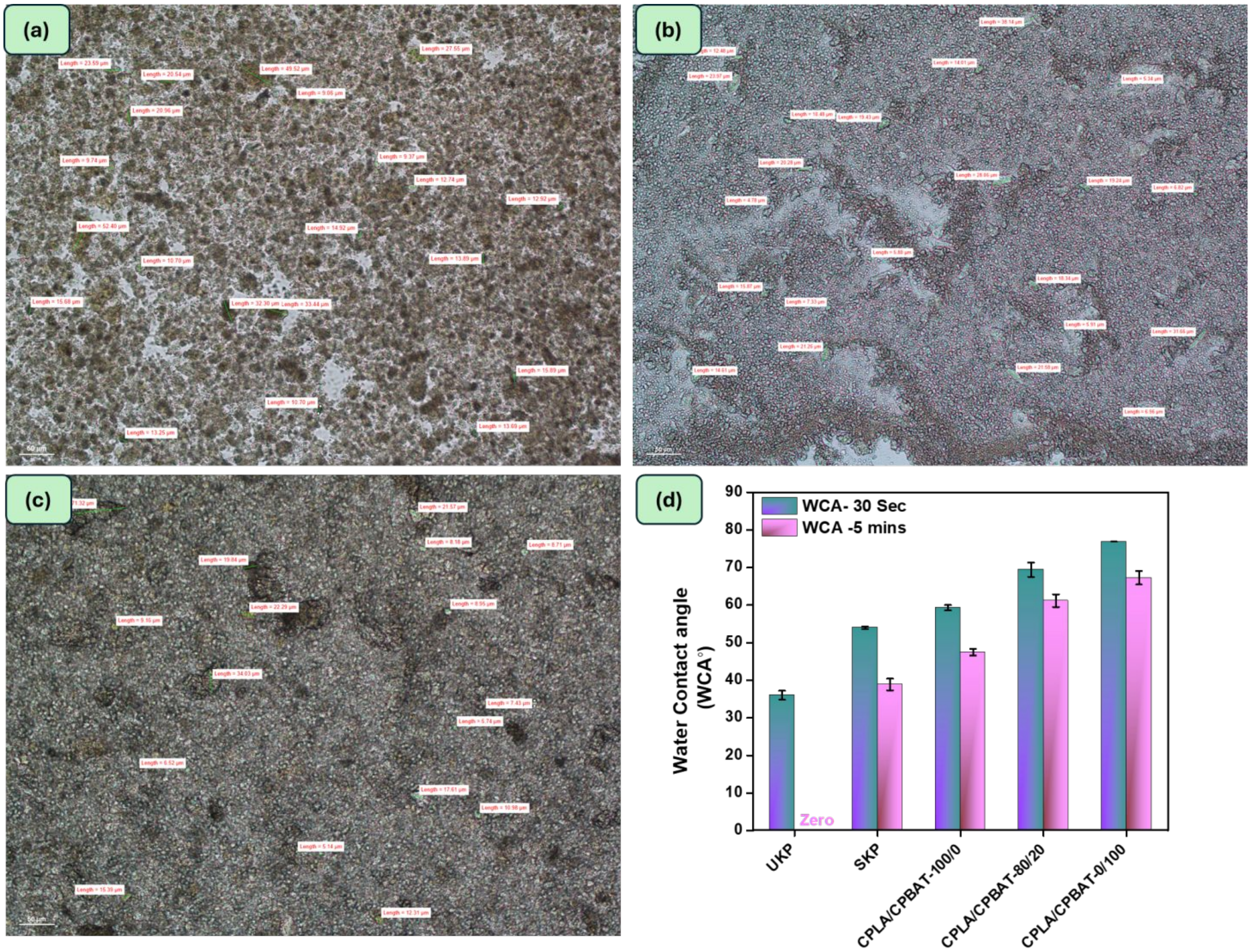

Figure S2. Brightfield-transmitted light, magnified images: (a) emulsion of CPLA only (CPLA/CPBAT-100/0), (b) emulsion of CPBAT only (CPLA/CPBAT-0/100); and (c) emulsion of blend of CPLA/CPBAT (PLA/CPBAT-50/50). Water contact angle data for unmodified coated paper and various coated paper samples (d). The water contact angles were measured 30 s and 5 min after applying each water droplet onto the surface of a sample.

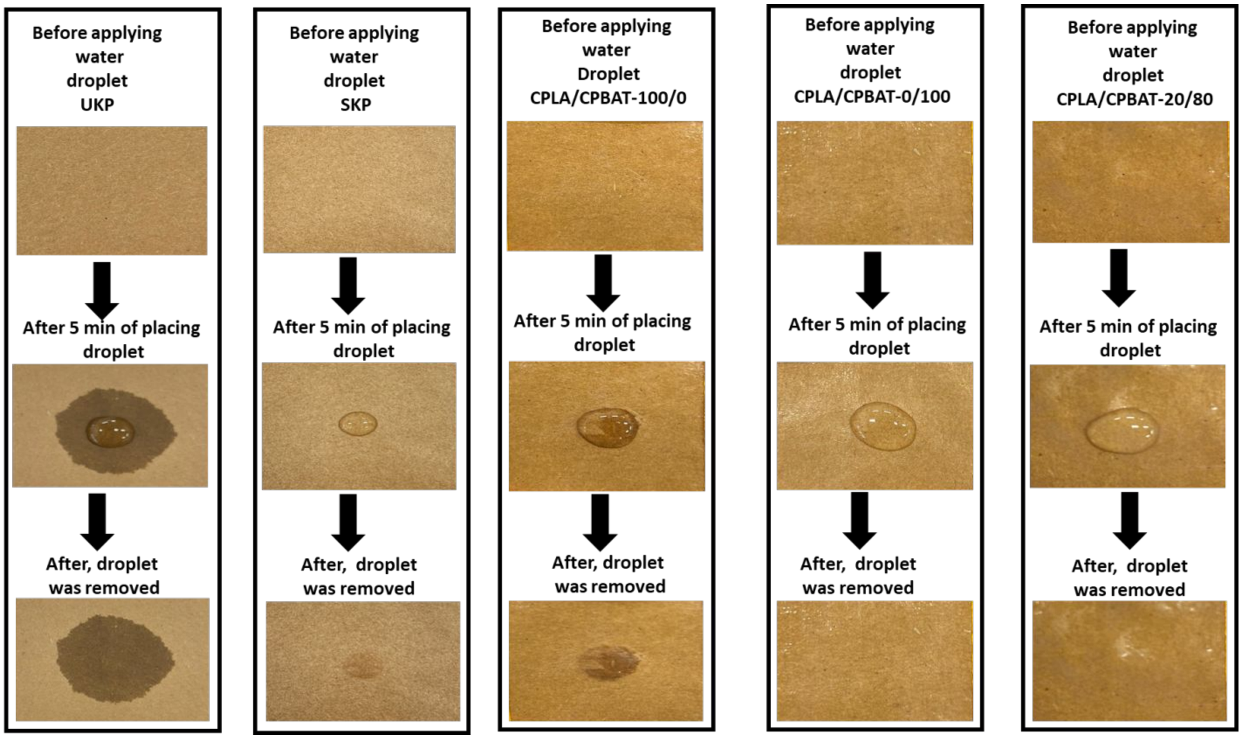

Figure S3. Images showing unmodified kraft paper (UKP), as well as various coated paper samples including, SKP, CPLA/CPBAT-100/0, CPLA/CPBAT-0/100, and CPLA/CPBAT-20/80. These photographs were taken before and after water droplets were applied, five minutes after the droplets were applied, and after the droplets were removed.

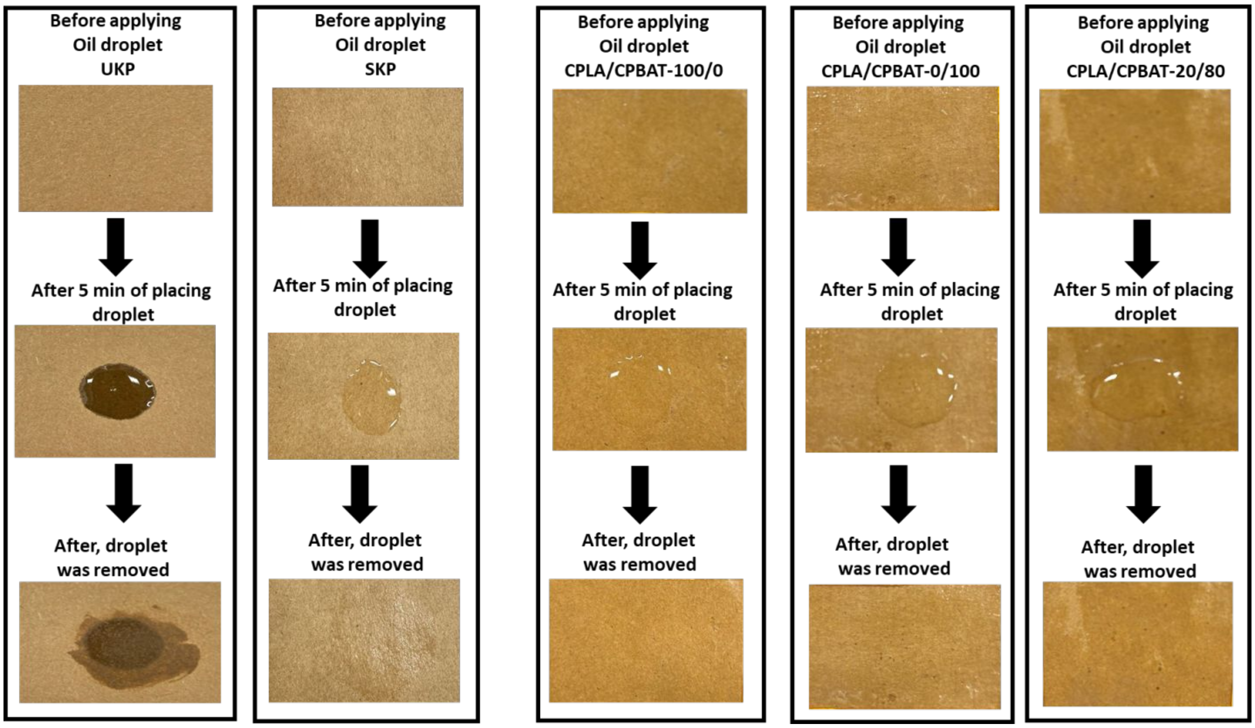

Figure S4. Images showing unmodified kraft paper (UKP), as well as various coated paper samples including, SKP, CPLA/CPBAT-100/0, CPLA/CPBAT-0/100, and CPLA/CPBAT-20/80. These photographs were taken before castor oil droplets were applied, five minutes after the droplets were applied, and after the droplets were removed.

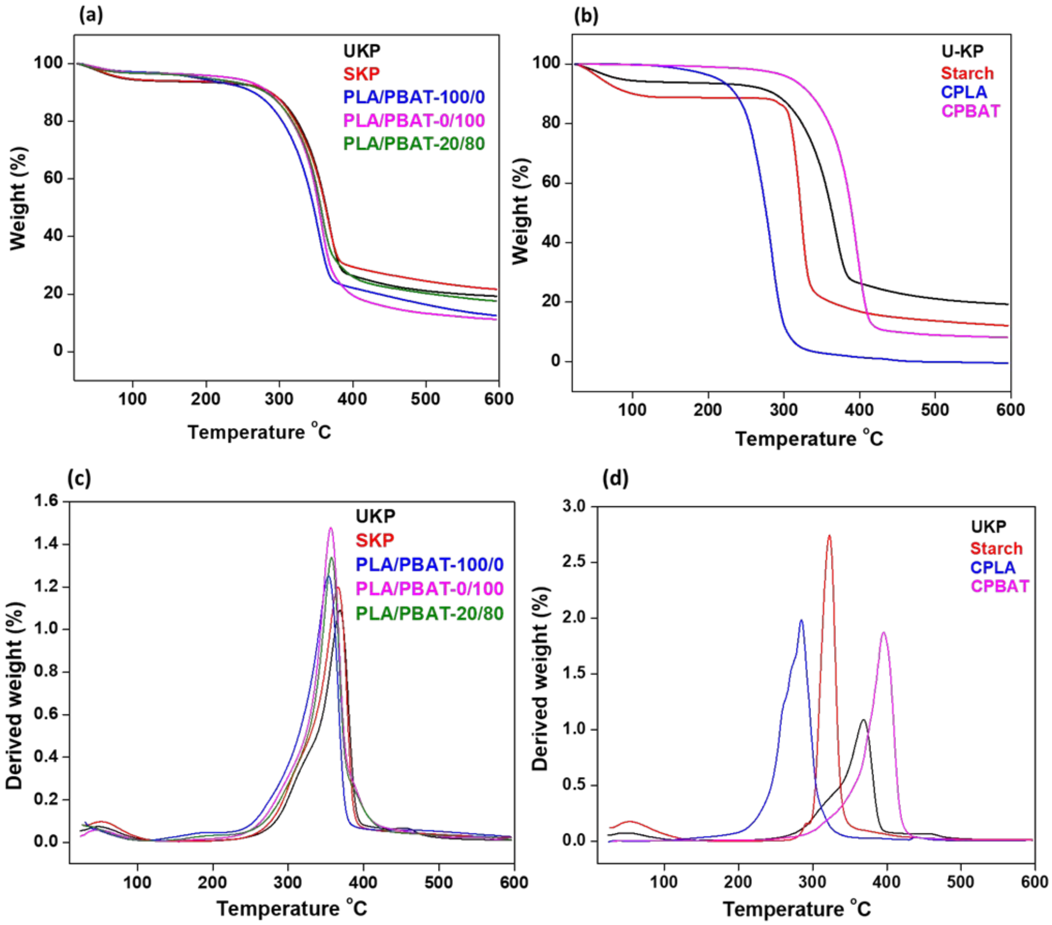

Figure S5. TGA plots of (a) unmodified kraft paper and all coated paper samples and (b) TGA plots of unmodified kraft paper and the coating materials. DTG plots of (c) unmodified kraft paper and all coated paper samples and (d) DTG plots of coating materials.

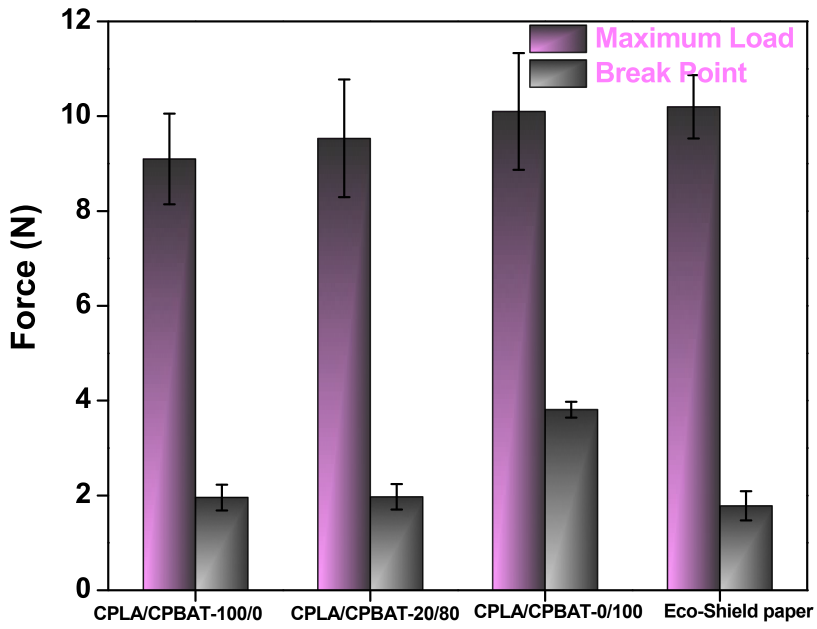

Figure S6. Seal strength values measured as force (N) required to break the seal at maximum load and break point for various paper samples, including PLA/CPBAT-100/0, CPLA/CPBAT-20/80, CPLA/CPBAT-0/100 compared to that of a commercial control (i.e., ECO-Shield paper).

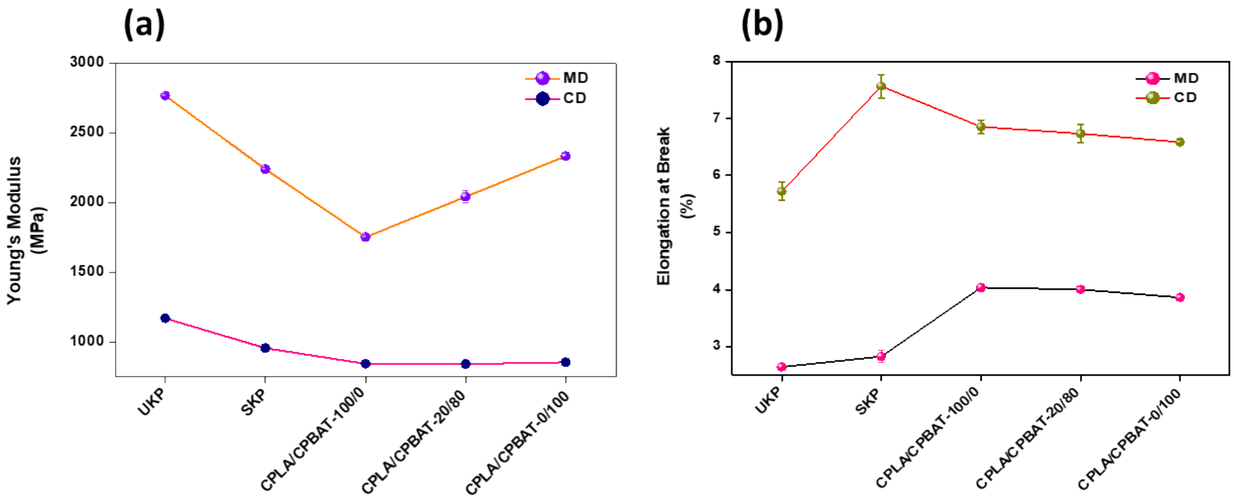

Figure S7. Young's modulus (a) and elongation at break (b) of unmodified paper kraft paper in comparison to various coated paper samples.

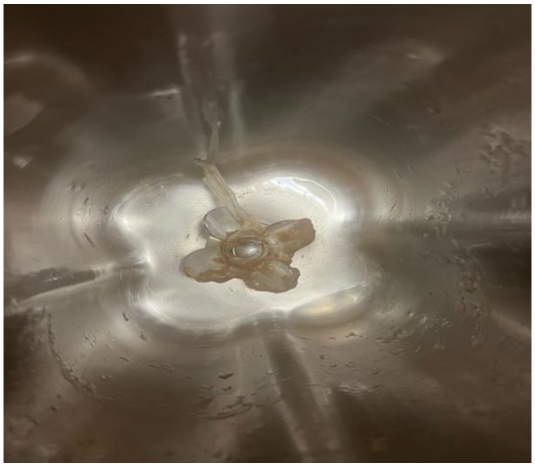

Figure S8. Photograph showing failure of the repulping test with commercial PBAT-coated paper. Plastic-like films had become wrapped around the blender.

Table S1. Carbon content of samples evaluated during biodegradation.

**Sample**	**Average Carbon content (%)**
Cellulose	42.5
UKP	40.2
CPBAT	43.26
CPLA/CPBAT-0/100	40.36
CPLA	50.51
CPLA/CPBAT-100/0	42.56
CPLA/CPBAT-50/50	41.53

Table S2 below presents the physicochemical analysis for compost performed by an external lab. Information about the procedure is provided in the links at the end of the table.

Table S2. Physicochemical parameters of compost used for biodegradation test

**Tests**	**Units**	**Desired Range**	**Results**
pH		6.0 – 8.0	7.22
E. C. - Saturation Paste	mmho/cm	< 4	13.8
Nitrate-N (NO_3-N)[1]	ppm	40 – 99	

Ammonium-N (NH_4-N)[1]	ppm		
Total Dry Solid	%		55.7
Total Volatile Solid	%		44.4
C/N Ratio		<25	10.3
Tests	Units		Wet Weight Basis
Total Nitrogen (N)	%		2.27
Total Phosphorus (P)	%		1.36
Total Potassium (K)	%		1.46
Total Calcium (Ca)	%		6.11
Total Magnesium (Mg)	%		1.57
Total Zinc (Zn)	ppm		380
Total Iron (Fe)	ppm		7033
Total Manganese (Mn)	ppm		294
Total Copper (Cu)	ppm		126
Total Carbon (C)	%		23.3
Total Sodium (Na)	%		0.347
Total Aluminum (Al)	%		0.198
Total Sulfur (S)	%		0.505
Total Boron (B)	ppm		39

Interpretation for nitrate-N is for growing media only. If this material is to be used as soil amendment, the interpretation for nitrate-N is not applicable.

[2].https://extension.missouri.edu/programs/soil-and-plant-testing-laboratory/spl-compost-analysis

[3].https://extension.missouri.edu/media/wysiwyg/Extensiondata/Pro/SoilAndPlantTestingLab/Docs/CompostAnalysisMethods.pdf

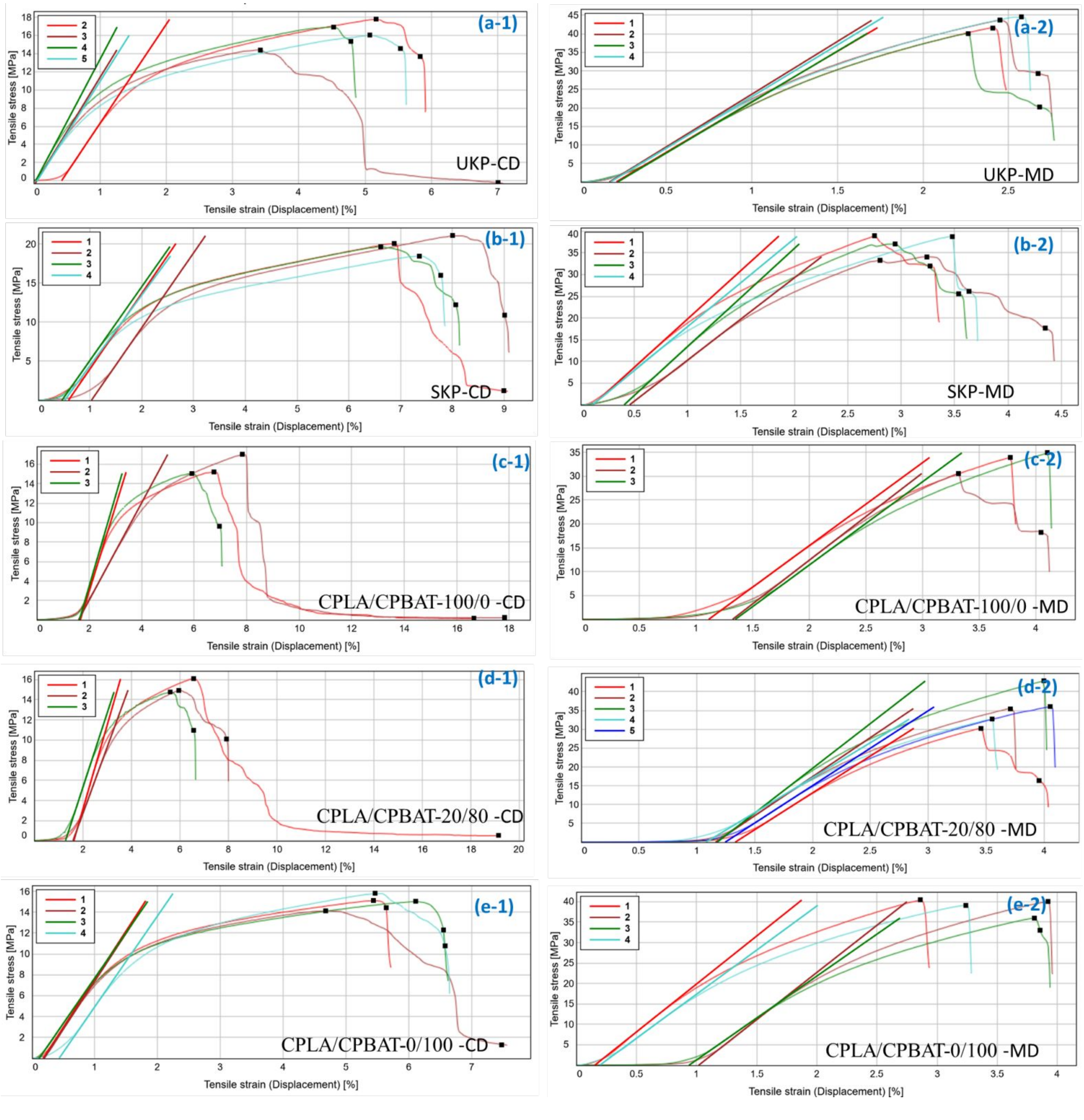

Figure S9. Stress-strain curves for uncoated and coated paper samples including UKP (a-1) in CD and (a-2) in MD, SKP (b-1) in CD and (b-2) in MD, CPLA/CPBAT-100/0 (c-1) in CD and (c-2) in MD, CPLA/CPBAT-20/80 (d-1) in CD and (d-2) in MD, CPLA/CPBAT-0/100 (e-1) in CD and (e-2) in MD.

Differential Scanning Calorimetry (DSC)

The thermal properties of the synthesized polymers CPBAT and CPLA were established by studying their melting temperature (T_m) and glass transition temperature (T_g) with the help of DSC analysis. The DSC thermograms of CPBAT and CPLA are shown in Figure S8. The analysis was carried out under cryogenic conditions beginning at a temperature of -50 °C. All the samples were studied under two heating cycles such as heating-cooling-heating cycles. The T_g of CPBAT was found to be ~-24 ℃, while the T_m was observed at ~ 115 ℃ for the CPBAT polymer, while for CPLA the T_m was observed at ~ 125 ℃. The complete thermal analysis for CPBAT and CPLA is shown in Figure S10, SI.

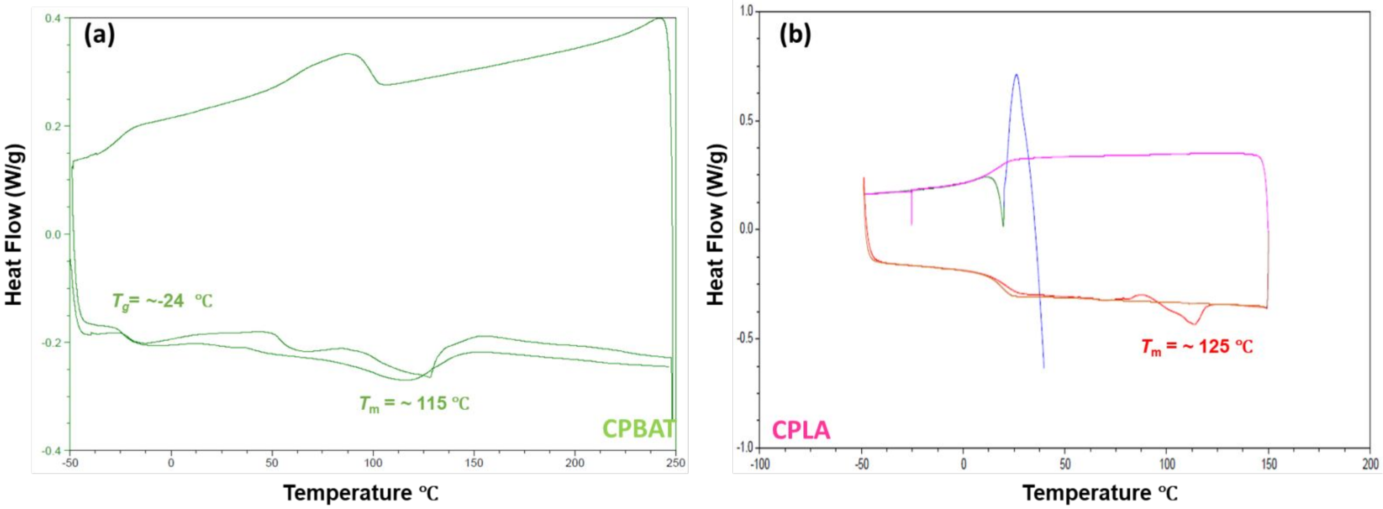

Figure S10. DSC analysis of the synthesized (a) CPBAT and (b) CPLA.

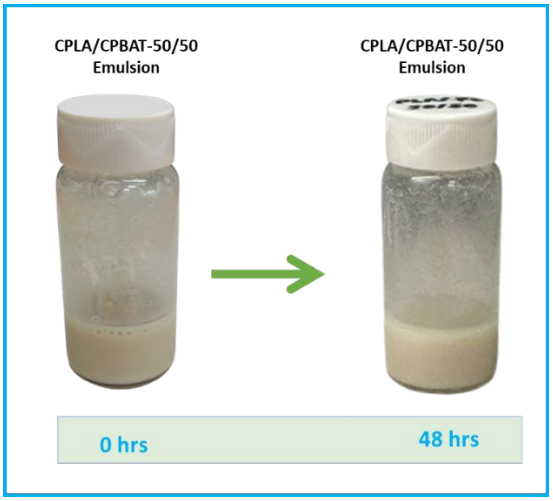

Figure S11. Stability of the CPLA/CPBAT-50/50 emulsion as demonstrated by capturing images of the emulsion at 0 and 48 h after emulsion formation.
